# The Metabolite Indole‐3‐Acetic Acid of *Bacteroides Ovatus* Improves Atherosclerosis by Restoring the Polarisation Balance of M1/M2 Macrophages and Inhibiting Inflammation

**DOI:** 10.1002/advs.202413010

**Published:** 2025-01-22

**Authors:** Wu Liu, Jingyu Wang, Heng Yang, Congcong Li, Wanqi Lan, Tingtao Chen, Yanhua Tang

**Affiliations:** ^1^ Department of Cardiovascular Surgery The Second Affiliated Hospital Jiangxi Medical College Nanchang University Nanchang 330008 China; ^2^ The Second Clinical Medical College of Nanchang University Nanchang 330008 China; ^3^ Department of Hematology The Second Affiliated Hospital of Nanchang University Nanchang 330008 China; ^4^ The Institute of Translational Medicine Jiangxi Medical College Nanchang University Nanchang 330036 China; ^5^ Jiangxi Province Key Laboratory of Bioengineering Drugs School of Pharmacy Jiangxi Medical College Nanchang University Nanchang 330036 China

**Keywords:** atherosclerosis, gut metabolites, gut microbiota, inflammation, macrophage polarisation

## Abstract

Emerging research has highlighted the significant role of the gut microbiota in atherosclerosis (AS), with microbiota‐targeted interventions offering promising therapeutic potential. A central component of this process is gut‐derived metabolites, which play a crucial role in mediating the distal functioning of the microbiota. In this study, a comprehensive microbiome‐metabolite analysis using fecal and serum samples from patients with atherosclerotic cardiovascular disease and volunteers with risk factors for coronary heart disease and culture histology is performed, and identified the core strain *Bacteroides ovatus* (*B. ovatus*). Fecal microbiota transplantation experiments further demonstrated that the gut microbiota significantly influences AS progression, with *B. ovatus* alone exerting effects comparable to volunteer feces from volunteers. Notably, *B. ovatus* alleviated AS primarily by restoring the intestinal barrier and enhancing bile acid metabolism, particularly through the production of indole‐3‐acetic acid (IAA), a tryptophan‐derived metabolite. IAA inhibited the TLR4/MyD88/NF‐κB pathway in M1 macrophages, promoted M2 macrophage polarisation, and restored the M1/M2 polarisation balance, ultimately reducing aortic inflammation. These findings clarify the mechanistic interplay between the gut microbiota and AS, providing the first evidence that *B. ovatus*, a second‐generation probiotic, can improve bile acid metabolism and reduce inflammation, offering a theoretical foundation for future AS therapeutic applications involving this strain.

## Graphical abstract

Briefly, patients with ASCVD have a reduced abundance of *B. ovatus* in their feces. Oral administration of live *B. ovatus* attenuated atherosclerosis. Specifically, *B. ovatus* restores the expression of intestinal barrier proteins and improves bile acid metabolism, while producing the tryptophan metabolite IAA restores the M1/M2 polarisation balance by inhibiting the M1 macrophage TLR4/MyD88/NF‐κB pathway and promoting M2 macrophage polarisation.

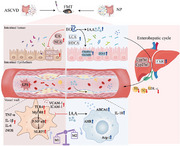

## 1 Introduction

Atherosclerotic cardiovascular disease (ASCVD) remains a leading global cause of morbidity and mortality: it is responsible for ≈17.7 million deaths each year, which represents 31% of total deaths throughout the world.^[^ ^1^ ^]^ Atherosclerosis (AS), characterized by endothelial dysfunction, chronic inflammation, and plaque formation in the arterial walls, is the primary driver of ASCVD.^[^ ^2^ ^]^ Although statins are the most widely used treatment to lower blood lipid levels and prevent complications, their efficacy is highly variable across individuals.^[^ ^3^, ^4^ ^]^ In cases where statin treatments fail, more invasive interventions, such as percutaneous coronary intervention, coronary artery bypass grafting, and endarterectomy, are required, but these procedures carry an increased risk of thrombosis.^[^ ^5^ ^]^ While conventional approaches targeting blood lipids and glucose metabolism have shown some benefits, they have not effectively reversed the rising trend of cardiovascular morbidity and mortality. Hence, it is crucial to explore novel therapeutic targets and metabolites to improve the diagnosis and treatment of ASCVD.

The risk factors that contribute to the development and progression of AS are multifaceted and include smoking, obesity, diabetes mellitus, hypertension, intestinal dysbiosis, physical inactivity, environmental stress, sleep disorders, and genetic predisposition.^[^ ^6^ ^]^ The pathogenesis of AS is complex, characterized by progressive inflammation marked by lipid accumulation, endothelial dysfunction, and cell death. Chronic inflammation is a key driver of recurrent AS lesions, even after significant reductions in cholesterol levels.^[^ ^7^, ^8^ ^]^ The balance between pro‐ and anti‐inflammatory mediators is essential for controlling AS progression and plaque stability.^[^ ^9^ ^]^ Macrophages play a central role in this process, acting as crucial mediators of immune responses. These cells exhibit remarkable plasticity with two major phenotypes: the pro‐inflammatory M1 type, which secretes inducible nitric oxide synthase (iNOS) and cytokines such as interleukin‐6 (IL‐6), and tumor necrosis factor‐alpha (TNF‐α) to promote tissue damage,^[^ ^10^ ^]^ and the anti‐inflammatory M2 type, which secretes factors including arginase‐1 (Arg‐1) and IL‐10 to facilitate tissue repair, to stabilize plaques, and to suppress pro‐inflammatory cytokines.^[^ ^11^ ^]^ Dong et al.^[^ ^12^ ^]^ recently demonstrated that gamma‐aminobutyric acid (GABA) activates the Janus kinase 1 (JAK1)/signal transducer and activator of transcription 6 (STAT6) pathway in macrophages, which inhibits the JAK2/STAT3 and nuclear factor kappa‐B (NF‐κB) pathways, promotes the M2 phenotype, and alleviates atherosclerotic inflammation. Thus, therapeutic strategies aimed at promoting M2 polarisation while inhibiting M1 polarisation are crucial for improving outcomes in AS.

The gut microbiota has garnered increasing attention for its role in modulating human health, particularly its influence on the progression of AS through various mechanisms. Gut‐derived metabolites, including trimethylamine‐N‐oxide (TMAO), short‐chain fatty acids (SCFAs), and secondary bile acids, are transported via the portal vein into the systemic circulation, where they function as signaling molecules that influence host metabolism and immune responses.^[^ ^13^, ^14^ ^]^ Recent studies suggest that reducing TMAO levels promotes M2 polarisation and enhances the stability of atherosclerotic plaques.^[^ ^15^ ^]^ Ma et al.^[^ ^16^ ^]^ demonstrated that butyrate, a key SCFA, mitigates chronic atherosclerotic inflammation by inhibiting M1 polarisation through the histone deacetylase (HDAC)/Sp1/peroxisome proliferator‐activated receptor gamma (PPARγ)/NF‐κB or NOD‐, LRR‐, and pyrin domain‐containing protein 3 (NLRP3) signaling pathway. Furthermore, *Lactobacillus plantarum* ATCC 14 917 has been shown to exert anti‐atherosclerotic effects by modulating pro‐inflammatory cytokines and oxidative stress.^[^ ^17^ ^]^ Conversely, *Fusobacterium nucleatum* ATCC 25 586 exacerbates AS by promoting M1 polarisation and disrupting lipid metabolism.^[^ ^18^ ^]^ However, the role of indole metabolites derived from dietary amino acids in AS pathogenesis has been underexplored, and their direct in vivo effects remain poorly understood.

In this study, we performed a comprehensive microbiome‐metabolite analysis of fecal and serum samples from patients with ASCVD and volunteers with risk factors for coronary heart disease to investigate the clinically relevant relationship between gut microbiota and ASCVD. Additionally, we employed a high‐fat diet (HFD)‐induced apolipoprotein E knockout (*Apoe* ^−/−^) mouse model of AS, along with an M1/M2 macrophage polarisation model, to explore the underlying mechanisms. We found that *Bacteroides ovatus* (*B. ovatus*) has a beneficial impact on AS, reducing inflammation in mice by restoring the intestinal barrier, enhancing bile acid and lipid metabolism, and, notably, producing indole‐3‐acetic acid (IAA). This metabolite inhibited the M1 macrophage Toll‐like receptor 4 (TLR4)/myeloid differentiation primary response protein 88 (MyD88)/NF‐κB signaling pathway and promoted M2 macrophage polarisation, thereby restoring the balance between M1 and M2 macrophages. These results provide novel insight into the interplay between gut microbes and AS, demonstrating, for the first time, that *B. ovatus*, a second‐generation probiotic, can improve lipid metabolism and reduce inflammation. This provides a theoretical foundation for future therapeutic applications involving this strain in AS and the development of related formulations.

## 2 Results

### 2.1 *B. ovatus* Is a Key Bacterium in the Gut Microbiota of Volunteers with Risk Factors for Coronary Heart Disease

We recruited 30 patients with ASCVD and 30 volunteers with risk factors for coronary heart disease (the NP group) to investigate the impact of the gut microbiota on AS. The baseline characteristics and clinical data of the participants are summarised in **Table** **1**. There were no significant differences between the 2 groups in terms of age, body mass index (BMI), blood pressure, history of chronic diseases, medication use, and levels of high‐density lipoprotein cholesterol (HDL‐c) and LDL‐c. However, the ASCVD group had significantly higher plasma total cholesterol (TC) (*p* < 0.001) and triglycerides (TG) (*p* = 0.003) levels. Notably, cardiac troponin I (cTnI) levels were significantly elevated in the ASCVD group compared with the NP group (*p* < 0.001), indicating heart damage in the patients with ASCVD.

**Table 1:** Basic Characteristics of the Study Participants. Normally distributed continuous variables are reported as mean ± standard deviation (SD), non‐normally distributed variables are expressed as the median (interquartile range), and categorical variables are reported as numbers and percentages. All tests were statistically significant with a two‐tailed *p*‐value < 0.05. Differences between groups were compared using the independent samples chi‐square (χ^2^) test for categorical variables because they were non‐hierarchical. Normally distributed variables were compared with the independent samples t‐test, and non‐normally variables distributed were compared with the independent samples Mann – Whitney U test. Abbreviations: BMI, body mass index; CHD, coronary heart disease; Number of bypass vessels, coronary artery bypass grafting; TC, total cholesterol; TG, triglycerides; HDL‐c, high‐density lipoprotein cholesterol; LDL‐c, low‐density lipoprotein cholesterol; WBC, white blood cell.

Variables	ASCVD	NP	*p* Value
	N = 30	N = 30	
Age(y)	60(56–68.5)	59(45.5–72)	0.51
Female, n (%)	13 (43.3)	17 (56.7)	0.008
BMI (kg/m^2^)	24.175(23.085–25.238)	24.07(20.648–26.478)	0.723
Systolic pressure(mmHg)	132.5(123–160)	147.5(127.5–177.25)	0.099
Diastolic pressure(mmHg)	84.5(76.75–91)	88.5(83–96.25)	0.1
Pulse(bpm)	78.5(73–85.25)	78(72–82.25)	0.51
Hypertension, n (%)	13 (43.3)	14 (46.7)	0.795
Hyperglycemia, n (%)	7(23.3)	4 (13.3)	0.317
Hyperlipidemia, n (%)	10(33.3)	9 (30)	0.781
History of atherosclerosis, n (%)	17(56.7)	7 (23.3)	0.008
History of cancer, n (%)	1(3.3)	1 (3.3)	1
Smoking/drinking history, n (%)	11(36.7)	2 (6.7)	0.005
Family history of CHD, n (%)	1(3.3)	0 (0)	0.317
Statins drugs, n (%)	3(10)	1 (3.3)	0.29
Anti‐hypertensive drugs, n (%)	9(30)	8 (26.7)	0.774
Anti‐diabetic drugs, n (%)	3(10)	1 (3.3)	0.29
Gensini score	52(20–89.25)	NA	
Number of bypass vessels	3(1.75–3)	NA	
HDL‐c (mmol/L)	1.155±0.374	1.246±0.253	0.148
LDL‐c (mmol/L)	2.385(1.728–3.32)	2.39(1.985–2.775)	0.83
TC (mmol/L)	5.08(3.98–5.59)	4.1(3.34–4.685)	0.000
TG (mmol/L)	2.39±1.618	1.25±0.72	0.003
Fasting glucose(mmol/L)	5.385(4.983–6.198)	5.3(4.82–6.555)	0.935
Uric acid(µmol/L)	413.55(340.3–508.01)	319.9(238.24–385.65)	0.001
Serum creatinine (mg/dL)	85.82±27.94	66.99±21.18	0.341
Cardiac troponin I(µg/L)	0.361±0.808	0.028±0.062	0.000
WBC (10^9^/L)	5.85(4.89–7.35)	6(5.148–7.283)	0.912

**Figure 1:**
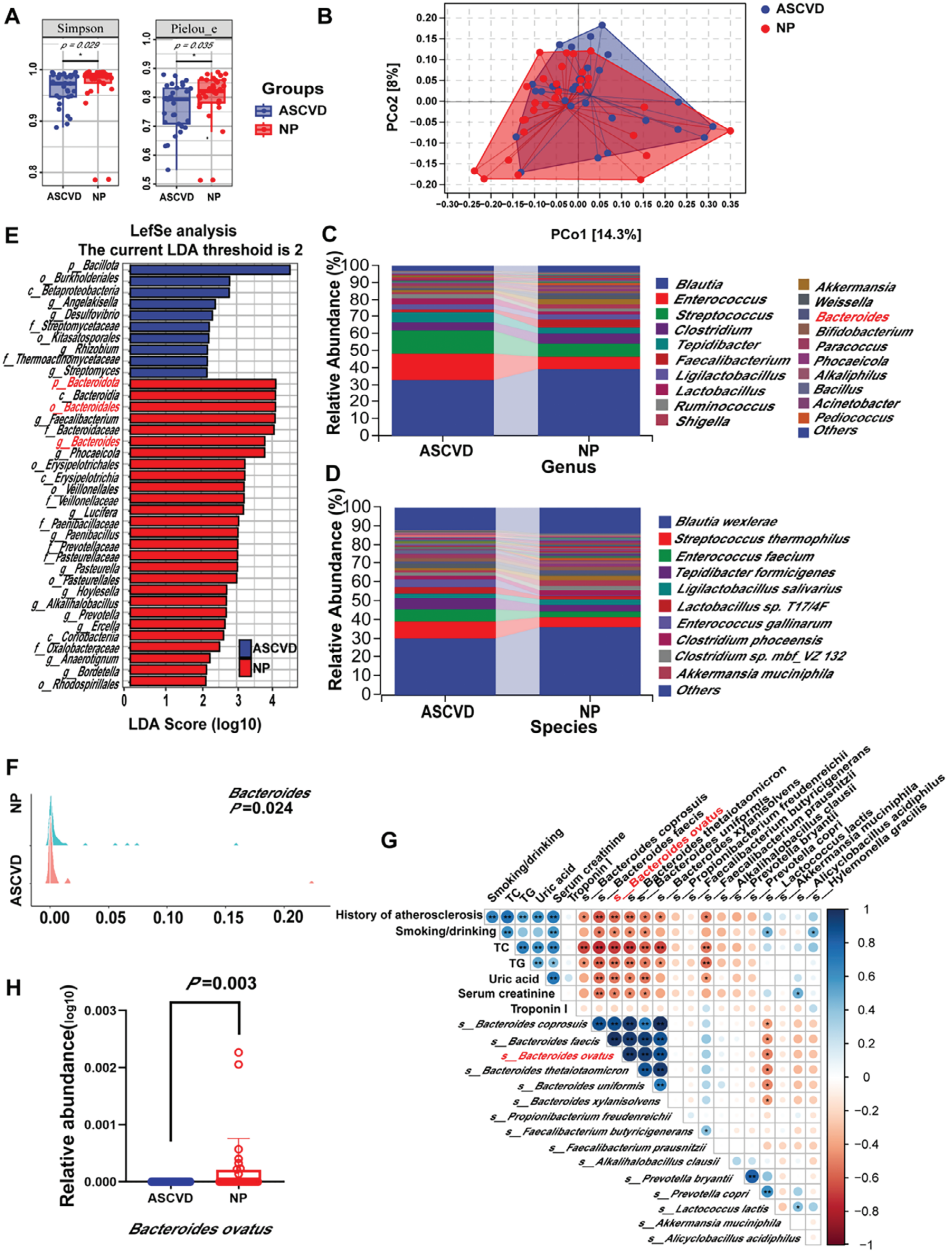
*B. ovatus* is a key bacterium in the gut microbiota of volunteers with risk factors for coronary heart disease. A) Alpha diversity of the microbial community. B) Differences in the microbial community composition (beta diversity). C) Genus classification horizontal histogram. D) Species classification horizontal histogram. E) Linear discriminant analysis (LDA) effect size of 16S ribosomal RNA (rRNA) gene sequences generated histograms, relative abundance of bacteria (LDA > 2). F) Map of the *Bacteroides* genus level mountain ranges, *n* = 30. G) Differential clinical features and intestinal microbial correlation heat map. Blue indicates positive correlations and red indicates negative correlations. H) *B. ovatus* relative abundance, *n* = 30. Summary data are presented as the mean ± standard deviation. Statistical significance was determined by the Mann‐Whitney U test (F, H). *p < 0.05. Abbreviations: ASCVD, atherosclerotic cardiovascular disease. NP, control population. TC, total cholesterol. TG, triglycerides. PCo, Principal coordinate.

We performed full‐length 16S ribosomal RNA (rRNA) gene sequencing (regions V1‐V9) in fecal samples to explore differences in the gut microbiota composition. There was analysis revealed significantly lower α‐diversity in the ASCVD group compared with the NP group, as indicated by the Simpson and Shannon diversity indices, Pielou's evenness homogeneity, and the Chao1 richness index (**Figure** **1**A; Figure S1A, Supporting Information). There was also a significant difference in β‐diversity analysis between the groups (Figure 1B). A genus‐level classification histogram (Figure 1C) and a mountain range diagram visualizing common fungal genera (Figure S1C, Supporting Information) further highlighted distinct differences in the community composition between the groups. The species‐level classification results corroborated these findings (Figure 1D). Linear discriminant analysis (LDA) distribution histograms for significantly different species (Figure 1E) and the corresponding cladogram (Figure S1B, Supporting Information) indicated that beneficial bacteria, such as *Bacteroides*, *Clostridium*, and *Bacillus*, were enriched in the NP, while harmful bacteria, including *Proteobacteria*, *Betaproteobacteria*, *Desulfovibrio*, *Streptomyces*, and *Thermoactinomycetes*, were more prevalent in the ASCVD group. The relative abundance of food‐usable strains was consistently higher in the NP group (Figure S1D, Supporting Information). Together, these analyses confirm significant differences in the structural composition of the microbiota between the ASCVD and NP groups.

To identify key bacterial strains influencing ASCVD, we performed a detailed compositional analysis of the gut microbiota. There were significant differences were observed at the genus level, particularly for *Bacteroides* (*p* = 0.024; Figure 1F). A stacked histogram illustrating the distribution of *Bacteroides* species (Figure S1E, Supporting Information), and relative abundance analyses (Figure S1F, Supporting Information) showed that *B. ovatus* was significantly more abundant in the NP group compared with the ASCVD group (*p* = 0.003). Correlation analyses between food‐usable strains, *Bacteroides* species, and clinical characteristics (Figure 1G) revealed that *B. ovatus* correlated negatively with a history of AS, smoking/drinking habits, uric acid levels, serum creatinine, troponin I, TC, and TG. Additionally, *B. ovatus* correlated positively with the presence of certain probiotics. These findings suggest a dysbiosis in the ASCVD group compared with the NP group. At the same time, *B. ovatus* was enriched in the NP group (*p* = 0.003; Figure 1H), suggesting that *B. ovatus* may be a potential probiotic for ASCVD.

### 2.2 Human Fecal Microbiota Transplantation (FMT) Modulates AS Development in *Apoe* ^−/−^ Mice

**Figure 2:**
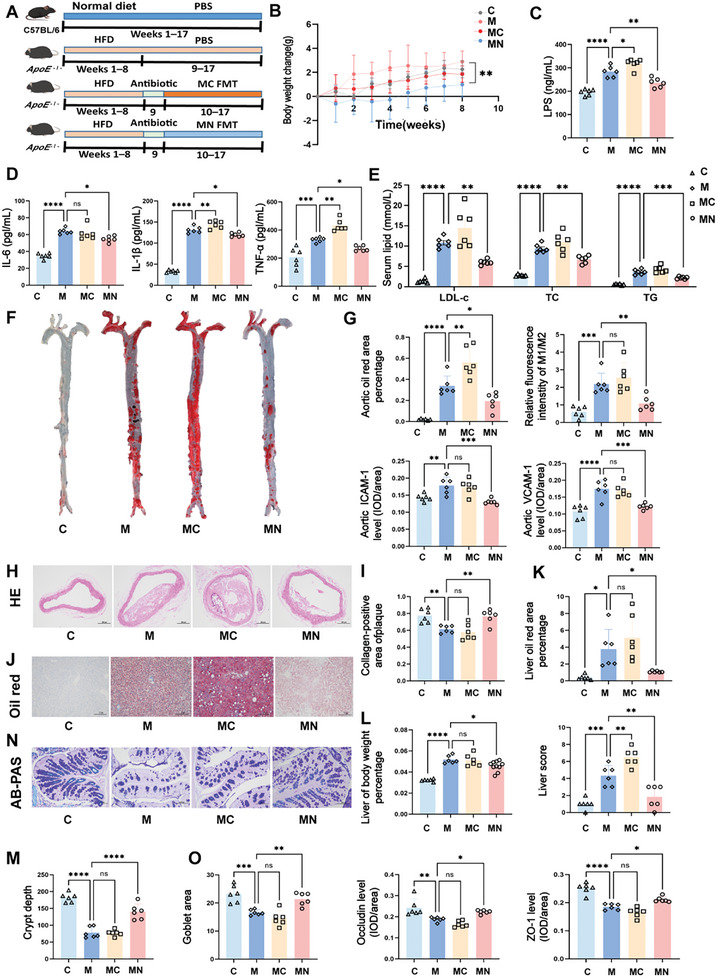
Human FMT modulates AS development in *Apoe* ^−/−^ mice. A) Experimental design: C57BL/6J mice were fed a normal diet (ND), and *Apoe* ^−/−^mice were fed an HFD for 8 weeks, followed by intragastric administration of broad‐spectrum antibiotics (Abx) for 1 week, and experimental intervention with 200 µL of a fecal suspension every 2 days for 8 weeks via oral gavage). B) Weight change, *n* = 6‐10 mice/group. C, D, E) Serum LPS levels, IL‐6, IL‐1β, and TNF‐α levels, cholesterol content (LDL‐c, TC, and TG levels), *n* = 6. F) Representative image of an oil red “O” stained aorta. G) Percentage of aortic oil red “O” stained area, the aortic immunofluorescence macrophage 1/macrophage 2 ratio, IHC of ICAM‐1, VCAM‐1, *n* = 6. H) Representative image of HE‐stained aorta (Scale bar: 200 µm). I) Collagen quantification of aortic's trichrome Masson staining, *n* = 6. J) A representative image of oil red “O”‐stained Liver (Scale bar: 200 µm). K) Percentage of the liver oil red “O”‐stained area, *n* = 6. L) Liver weight (*n* = 6–12), and liver score (*n* = 6). M) Colonic crypt depth, *n* = 6. N) Colon AB‐PAS staining (Scale bar: 100 µm). O) The number of goblet cells, IHC of occludin, and ZO‐1 in the colon, *n* = 6. Summary data are presented as the mean ± SEM. Statistical significance was determined using one‐way analysis of variance (ANOVA) followed by Dunnett's multiple comparison test for group comparisons. ns, not significant, *p < 0.05, **p < 0.01, ***p < 0.001, ****p < 0.0001. Abbreviations: C, control group. M, HFD‐fed *Apoe* ^−/−^ mice. MC, HFD‐fed *Apoe* ^−/−^ mice receiving fecal transplants from patients with ASCVD. MN, HFD *Apoe* ^−/−^ mice receiving fecal transplants from volunteers with risk factors for coronary heart disease. LPS, lipopolysaccharide. IL‐6, inteleukin‐6. TNF‐α, tumor necrosis factor‐alpha. LDL‐c, low‐density lipoprotein cholesterol. TC, total cholesterol. TG, total triglyceride. ICAM‐1, intercelladhesion molecule‐1. VCAM‐1, vascular cell adhesion molecules‐1. ZO‐1, zonula occludin 1. AB‐PAS, Alcian Blue‐Periodic Acid Schiff. IHC, immunohistochemical. HE, haematoxylin, and eosin. FMT, Fecal Microbiota Transplantation.

To investigate the role of the gut microbiota in the development of AS, we determined whether the effects observed in the clinical populations could be replicated in animal models. HFD‐fed *Apoe* ^−/−^ mice underwent FMT with feces from the ASCVD patients or NP group (**Figure** **2**A). The HFD‐fed *Apoe* ^−/−^ mice (M group) exhibited significant weight gain compared with the control (C) group, a trend that was mitigated following FMT from the volunteers with risk factors for coronary heart disease (MN group) (*p* = 0.0016; Figure 2B). Serum levels of lipopolysaccharide (LPS) (Figure 2C) and inflammatory markers, including IL‐6, TNF‐α, and IL‐1β (Figure 2D), indicated that HFD‐fed *Apoe* ^−/−^ mice subjected to FMT from patients with ASCVD (MC group) experienced a more pronounced inflammatory response than the M group. In contrast, the MN group exhibited significant reductions in TC (by 29%), LDL‐c (by 37%), and TG (by 39%) compared with the M group (Figure 2E). Aortic weight and anatomical assessments (Figure S2A,B, Supporting Information) revealed an increased plaque area in the MC group, while the MN group showed reduced aortic plaque formation. Specifically, aortic oil red “O” staining indicated a 20% increase in the plaque area in the MC group, whereas the MN group exhibited a 15% reduction (Figure 2F,G), as evidenced by representative plaque morphology in the aortic inner wall (Figure 2H). Further evaluation of the inflammation profile in the aorta revealed a 50% reduction in the M1/M2 macrophage ratio in the MN group compared with the M group, accompanied by decreased levels of inflammatory chemokines, intercell adhesion molecule‐1 (ICAM‐1), and vascular cell adhesion molecules‐1 (VCAM‐1), which were elevated in the MC group relative to the M group (Figure 2G; Figure S2C, Supporting Information). Masson's trichrome staining, a critical indicator of plaque stability, revealed a normal collagen content in the C group, decreased collagen in the M and MC groups, and a 26% increase in the MN group relative to the M group (Figure 1; Figure S2D, Supporting Information).

To examine the origins of lipid metabolism disturbances, we quantified liver fat deposition through oil red “O” staining (Figure 2J,K). The MN group exhibited a 70% reduction compared with the M group. There were also reductions in the liver solid weight and the liver scores (Figure 2L), highlighting the beneficial effects of FMT from the NP group on liver lipid metabolism. As the intestine is a primary target for FMT, we assessed its condition following this intervention. Measurements of the colon length (Figure S2G,F, Supporting Information) and crypt depth (Figure 2M), alongside histological analysis via HE staining (Figure S2H, Supporting Information), revealed that the M and MC groups had shallower and shorter colonic crypts. In contrast, the MN group exhibited significant improvements in these parameters, suggesting restored intestinal structure. Further analysis using Alcian Blue‐Periodic Acid Schiff (AB‐PAS) staining showed a decrease in goblet cells, indicative of impaired intestinal function, in the M and MC groups, while the MN group displayed an increase in the number of goblet cells (Figure 2N,O). Expression of intestinal permeability proteins, including zonula occludens 1 (ZO‐1) and occludin (Figure 2O), which are key markers of intestinal barrier integrity, was significantly enhanced in the MN group, indicating restoration of intestinal function disrupted by the HFD. Finally, fecal microbiota analysis post‐FMT (Figure S2M,N, Supporting Information) confirmed that, after antibiotic treatment, the humanized microbiota from both the ASCVD patients and NP groups effectively colonized the mouse intestines, validating the success of the FMT.

### 2.3 *B. ovatus* Regulates Bile Acid and Lipid Metabolism to Attenuate AS

**Figure 3:**
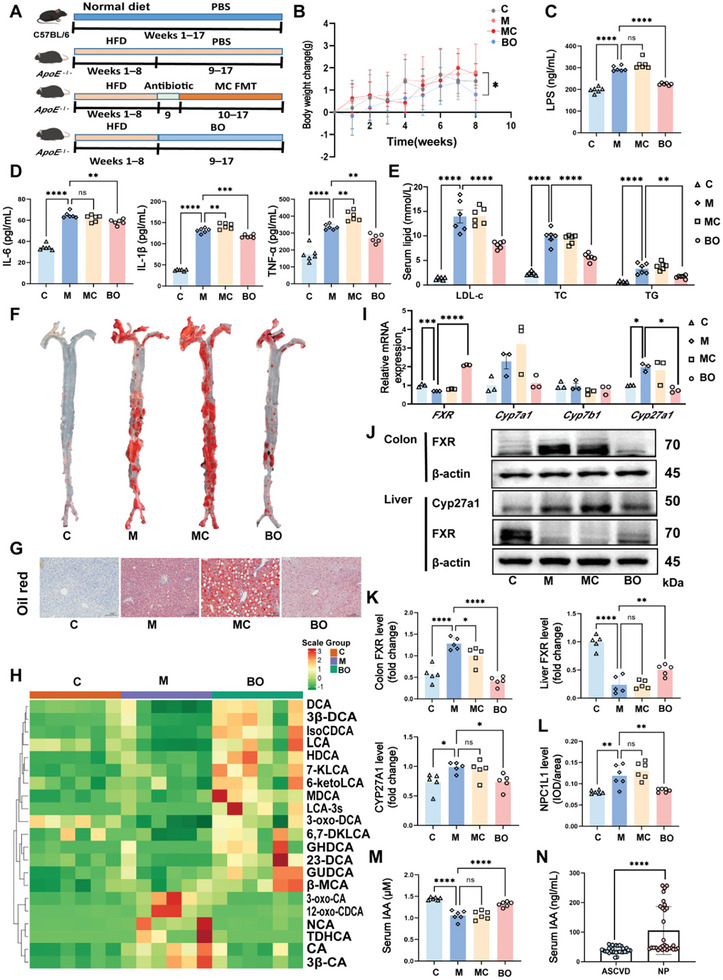
*B. ovatus* regulates bile acid and lipid metabolism to attenuate AS. A) Experimental design: C57BL/6J mice were fed a normal diet (ND), and *Apoe* ^−/−^ mice were fed a high‐fat diet (HFD) for 8 weeks, followed by intragastric administration of broad‐spectrum antibiotics (Abx) for 1 week. The experimental intervention was a fecal suspension of 200 µL of *B. ovatus* (1×109 colony‐forming units/mL) once every 2 days for 8 weeks via oral gavage. B) Weight change, *n* = 6–10 mice/group. C, D, E), Serum LPS levels, IL‐6, IL‐1β, and TNF‐α levels, cholesterol content levels, *n* = 6. F) Representative image of an oil red “O”‐stained aorta. G) Representative image of oil red “O”‐stained liver (Scale bar: 200 µm). H) Differential bile acid heat map in Feces. Red represents a positive correlation, and green represents a negative correlation, *n* = 6. I) Real‐time polymerase chain reaction detection of the liver genes. *FXR*, and *Cyp27a1*, *n* = 3. J) Representative western blot in colon and liver, *n* = 5. K) Relative protein expression of colon FXR, Liver FXR, and CYP27A1, *n* = 6. L) Quantitative immunohistochemical results of NPC1L1 in the colon, *n* = 6. M) Serum IAA levels, *n* = 6. N) Human serum IAA levels, *n* = 30. Summary data are presented as the mean ± SEM. Statistical significance was determined using one‐way analysis of variance (ANOVA) followed by Dunnett's multiple comparison tests for group comparisons and Mann‐Whitney U test (N). ns, not significant, *p < 0.05, **p < 0.01, ***p < 0.001, ****p < 0.0001. Abbreviations: BO, *B.ovatus*. IAA, indole‐3‐acetic acid. LPS, lipopolysaccharide. IL‐6, inteleukin‐6. TNF‐α, tumor necrosis factor‐alpha. LDL‐c, low‐density lipoprotein cholesterol. TC, total cholesterol. TG, total triglyceride. DCA, deoxycholic acid. LCA, lithocholic acid. CA, cholic acid. MCA, muricholic acid. TDHCA, Taurodehydrocholic acid. NCA, norcholic acid. FXR, farnesoid X receptor. Cyp7a1, cholesterol 7α‐hydroxylase. Cyp27a1, sterol 27‐hydroxylase. Cyp7b1, oxysterol 7a‐hydroxylase. NPC1L1, Niemann–Pick type C1‐like 1 protein. ASCVD, is atherosclerotic cardiovascular disease. NP, control population.

To explore the therapeutic potential of, *B. ovatus*, a key bacterium we identified in the gut microbiota of the NP group, we first evaluated its probiotic properties in vitro. Based on resistance assays (Figure S3C,D, Supporting Information) and bacteriostatic tests (Figure S3F,G, Supporting Information), *B. ovatus* exhibited partial resistance to antibiotics and significant inhibitory effects against common intestinal pathogens. Additionally, *B. ovatus* showed tolerance to bile salts (Figure S3E, Supporting Information) and strong adherence to intestinal epithelial cells (Figure S3H,I, Supporting Information), supporting its potential functional role in vivo. To assess its in vivo effects, we treated HFD‐fed *Apoe* ^−/−^ mice with *B. ovatus* (the BO group) (**Figure** **3**A). Compared with the M group, the BO group showed a significant reduction in body weight (*p* = 0.0384) (Figure 3B). Serum levels of LPS, inflammatory factors (Figure 3C,D), and lipids (Figure 3E) were notably improved in the BO group compared with the M and MC groups. Evaluation of aortic plaque formation, a critical hallmark of AS, revealed a 7% increase in the plaque area in the MC group, while the BO group exhibited a 16% reduction, based on oil red “O” staining (Figure 3F) and plaque area quantification (Figure S4A, Supporting Information). Similarly, liver oil red “O” staining (Figure 3G) and the liver scores (Figure S4D, Supporting Information) confirmed that *B. ovatus* reduced hepatic fat deposition and liver damage. Assessments of the colon length and crypt depth (Figure S4F,G, Supporting Information) indicated that *B. ovatus* colonization restored the colonic structure. Furthermore, intestinal integrity improved, as indicated by increased levels of barrier proteins ZO‐1 and occludin (Figure S4H,I, Supporting Information). Systemic inflammation was reduced in the BO group, as evidenced by improved spleen and epididymal weights (Figure S4J,K, Supporting Information). In vitro adhesion, assays confirmed *B. ovatus* attachment to intestinal cells, and quantitative real‐time polymerase chain reaction (qPCR) with *B. ovatus*‐specific primers verified successful colonization in the mouse intestine (Figure S4L, Supporting Information), substantiating its potential as a therapeutic agent against AS.

To further investigate the cholesterol‐lowering mechanism of *B. ovatus*, we performed bile acid‐targeted metabolomics on fecal samples from the C, M, and BO groups (Figure 3H). The BO group exhibited significantly elevated levels of beneficial bile acids, such as deoxycholic acid (DCA) and lithocholic acid (LCA), while reversing the HFD‐induced accumulation of harmful bile acids, including cholic acid (CA). Subsequently, we examined the expression of hepatic genes related to bile acid metabolism (Figure 3I). The M and MC groups showed reduced expression of *farnesoid X receptor* (*FXR)*, and increased expression of *cholesterol 7α‐hydroxylase* (*Cyp7a1)* and *sterol 27‐hydroxylase* (*Cyp27a1)*, whereas the BO group exhibited the opposite trends. Western blotting of liver and intestinal proteins corroborated these findings, showing the opposite expression patterns in the BO group compared to the M and MC groups (Figure 3J,K). Notably, *B. ovatus* inhibited Niemann–Pick type C1‐like 1 protein (NPC1L1), a key protein involved in intestinal bile acid transport (Figure 3L), suggesting that it reduces enterohepatic bile acid circulation and improves systemic cholesterol levels. While these findings underscore the role of *B. ovatus* in bile acid metabolism, chronic inflammation remains a significant driver of atherosclerotic lesions, even after substantial cholesterol reduction. *B. ovatus* produces bile salt hydrolase (BSH), an enzyme that converts conjugated bile acids to free bile acids, increasing bile acid excretion and reducing enterohepatic circulation, a phenomenon we confirmed in our study. It remains unclear how *B. ovatus* influences inflammation independently of cholesterol changes because these changes were not associated with improved inflammation. This led us to hypothesize that additional metabolites, beyond bile acids, contribute to the anti‐atherogenic effects of *B. ovatus*.

**Figure 4:**
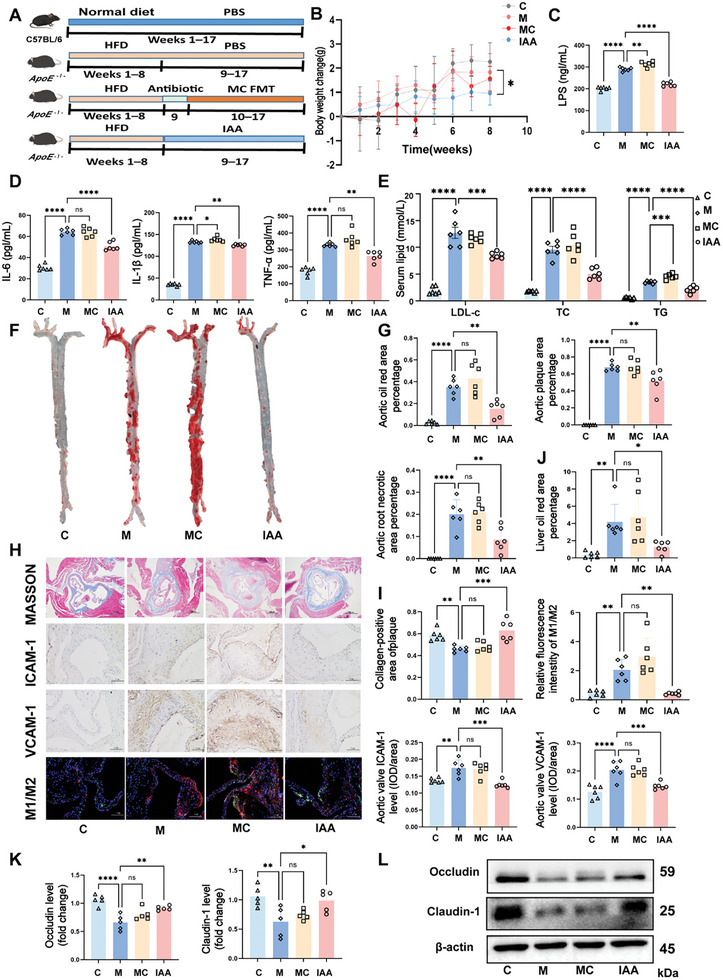
The role of *B. ovatus –* derived tryptophan metabolite IAA in attenuating AS. A) Experimental design: C57BL/6J mice were fed a normal diet (ND), and Apoe^−/−^ mice were fed a high‐fat diet (HFD) for 8 weeks, followed by intragastric administration of broad‐spectrum antibiotics (Abx) for 1 week. The experimental intervention was a fecal suspension or 200 µL of B. ovatus (1 × 109 colony‐forming units/mL) once every 2 days for 8 weeks via oral gavage. B) Weight change, *n* = 6–12 mice/group. C, D, E) Serum LPS levels, IL‐6, IL‐1β, and TNF‐α levels, cholesterol content levels, *n* = 6. F) Representative image of an oil red “O”‐stained aorta. G) The percentage of the aortic oil red “O”‐stained area, the plaque area, and the core area of plaque necrosis, *n* = 6. H) Representative images of Masson's trichrome staining (Scale bar: 500 µm), ICAM‐1 and VCAM‐1 immunohistochemistry (Scale bar: 100 µm), and the immunofluorescence M1/M2 diagram (Scale bar: 100 µm). I) Quantification of aortic Masson's trichrome staining, ICAM‐1, VCAM‐1, and the immunofluorescence M1/M2 ratio, *n* = 6. J) The liver oil red “O”‐stained area percentage, *n* = 6. K) Relative protein expression of occludin and claudin‐1 in the colon, *n* = 5. L) Representative occludin and claudin‐1 western blots. Summary data are presented as the mean ± SEM. Statistical significance was determined using one‐way analysis of variance (ANOVA) followed by Dunnett's multiple comparison test for group comparisons. ns, not significant, *p < 0.05, **p < 0.01, ***p < 0.001, ****p < 0.0001. Abbreviations: IAA, indole‐3‐acetic acid. LPS, lipopolysaccharide. IL‐6, inteleukin‐6. TNF‐α, tumor necrosis factor‐alpha. LDL‐c, low‐density lipoprotein cholesterol. TC, total cholesterol. TG, total triglyceride. ICAM‐1, intercelladhesion molecule‐1. VCAM‐1, vascular cell adhesion molecules‐1.

Recent studies have shown that *B. ovatus* can produce the tryptophan metabolite IAA, which activates the aryl hydrocarbon receptor (AHR) and mitigates HFD‐induced metabolic syndrome, hepatic steatosis, and associated inflammatory responses.^[^ ^29^ ^]^ Therefore, we assessed whether IAA also improves inflammation in the aortic wall. To explore this possibility, we performed a tryptophan assay on fecal samples and confirmed that *B. ovatus* indeed produces IAA in mice (Figure S4N, Supporting Information). Notably, IAA levels in feces (FigureS4O, Supporting Information) and serum (Figure 3M) were significantly elevated in the BO group compared with the M group. Importantly, IAA levels were also significantly higher in the NP group compared with the ASCVD group in clinical cohorts (Figure 3N), indicating that *B. ovatus* may play an important role in the modulation of AS through its production of IAA.

### 2.4 The Role of the *B. ovatus*‐Derived Tryptophan Metabolite IAA in Attenuating AS

To assess the therapeutic potential of IAA, a tryptophan metabolite produced by *B. ovatus*, we administered IAA to HFD‐fed *Apoe* ^−/−^ mice via oral gavage (**Figure** **4**A). IAA administration (the IAA group) resulted in a significant reduction in body weight compared with the M group (*p* = 0.0301; Figure 4B). Additionally, IAA treatment led to a decrease in serum LPS levels, reduced inflammatory cytokines (Figure 4C,D), and improved lipid profiles (Figure 4E), indicating its dual role in mitigating inflammation and regulating lipid metabolism. IAA treatment also attenuated AS progression, as evidenced by a significant reduction in the aortic solid weight (Figure S5A, Supporting Information) and a 20% decrease in the oil red “O”‐stained areas (Figure 4F,G). HE staining of the aortic valve revealed morphological changes in plaque characteristics, with quantification showing reductions in both the plaque area and the necrotic core size (Figure S4B, Supporting Information), suggesting that IAA contributes to plaque stabilization. Notably, there was a 37% increase in the aortic collagen content and substantial decreases in the VCAM‐1 (a 43% reduction) and ICAM‐1 (a 38% reduction) in the IAA group compared with the M group. Immunofluorescence staining of macrophage subtypes indicated a shift toward an anti‐inflammatory phenotype, with an improved M1/M2 balance (Figure 4H,I).

To further investigate the impact of IAA on lipid metabolism, we analyzed lipid profiles in associated organs. IAA treatment led to a significant reduction in the liver solid weight and the liver scores (Figure S5C, Supporting Information), along with a 70% reduction in liver lipid deposition, as indicated by oil red “O” staining (Figure 4J). These findings reinforce the conclusion that IAA effectively reverses hepatic lipid accumulation. Furthermore, IAA administration restored the colon length and crypt depth (Figure S5F, Supporting Information), while AB‐PAS staining showed an increase in the number of goblet cells and deeper crypts (Figure S5E, Supporting Information), suggesting improved intestinal morphology. Western blotting of key intestinal barrier proteins, including occludin and claudin‐1 (Figure 4K,L), demonstrated that IAA significantly restored their expression, and thus enhanced intestinal barrier integrity. Given these systemic improvements, we hypothesize that IAA enters the bloodstream and subsequently promotes vascular health. Serum analysis confirmed elevated IAA levels in treated mice (Figure S5I, Supporting Information), supporting the notion that IAA can directly influence the aorta and ameliorate systemic symptoms associated with AS.

**Figure 5:**
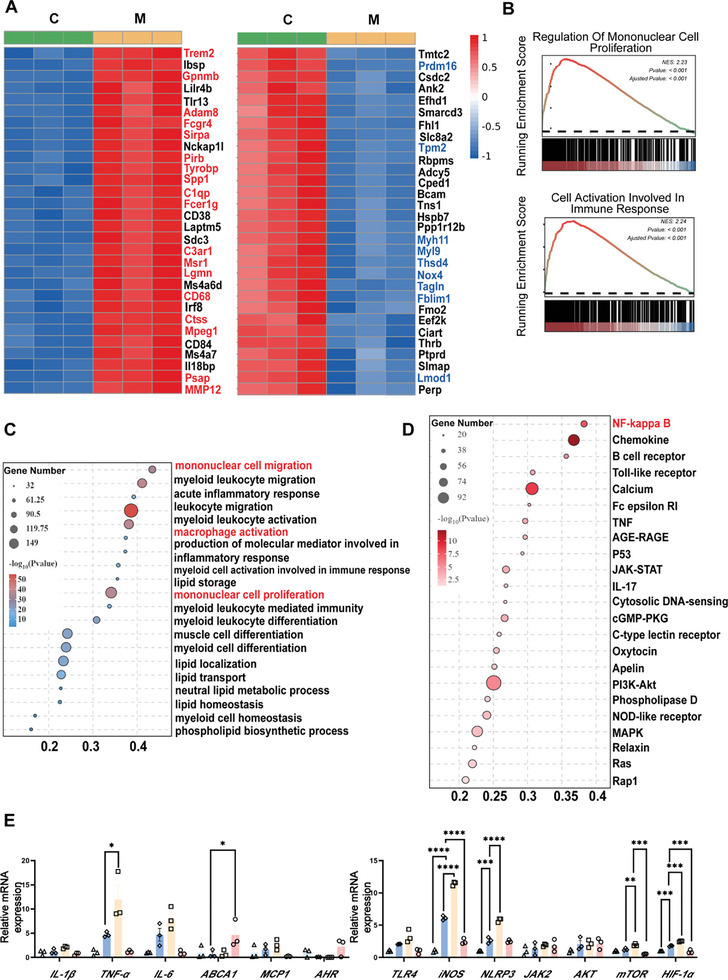
Aortic RNA‐seq to find the potential mechanism of AS. A) Aortic RNA‐seq differentially expressed gene heat map. Red represents a positive correlation, and blue represents a negative correlation. B) Gene ontology enrichment analysis bubble diagram, showing biological processes (BP). C) Kyoto Encyclopedia of Genes and Genomes bubble diagram. D) Gene Set Enrichment Analysis. E) Aortic gene expression. Summary data are presented as the mean ± SEM. Statistical significance was determined using one‐way analysis of variance (ANOVA) followed by Dunnett's multiple comparison test for group comparisons. *n* = 3 mice/group. *p < 0.05, **p < 0.01, ***p < 0.001, ****p < 0.0001. Abbreviations: *ABCA1*, ATP‐binding cassette transporter A1. *MCP1*, monocyte chemoattractant protein 1. *AHR*, aryl hydrocarbon receptor. *TLR4*, toll‐like receptor 4. *iNOS*, inducible nitric oxide synthase. *NLRP3*, NLR family pyrin domain containing 3. *JAK*, janus activated kinase. *AKT*, protein kinase B. *mTOR*, mammalian target of rapamycin. *HIF‐1α*, hypoxia‐inducible factor 1α.

RNA sequencing (RNA‐seq) of aortic tissue indicated substantial alterations in macrophage‐related gene expression in the top 30 upregulated Differentially expressed genes (DEGs) in HFD‐fed *Apoe* ^−/−^ mice. Additionally, genes related to smooth muscle cells and contractile function were altered in the top 30 downregulated DEGs (**Figure** **5**A). Increased expression of inflammatory markers such as IL‐1β and TNF‐α further underscored the critical role of inflammation in the pathogenesis of AS (Figure 5B; Figure S5J, Supporting Information). Gene Ontology (GO) enrichment analysis revealed that monocytes, the precursors of macrophages in atherosclerotic plaques, exhibited enhanced migration and differentiation pathways, along with increased macrophage activation (Figure 5C). Kyoto Encyclopedia of Genes and Genomes (KEGG) analysis further highlighted significant activation of the NF‐κB pathway, which provided the rationale for subsequent pathway validation (Figure 5D). QPCR revealed significant changes in the TLR4/MyD88/NF‐κB pathway and related inflammatory factors, but no notable alterations in lipid metabolism pathways, including protein kinase B (AKT)/mammalian target of rapamycin (mTOR)/hypoxia‐inducible factor 1‐alpha (HIF‐1α) and JAK (Figure 5E).

### 2.5 IAA Modulates Macrophage M1/M2 Polarisation to Reduce Inflammation

**Figure 6:**
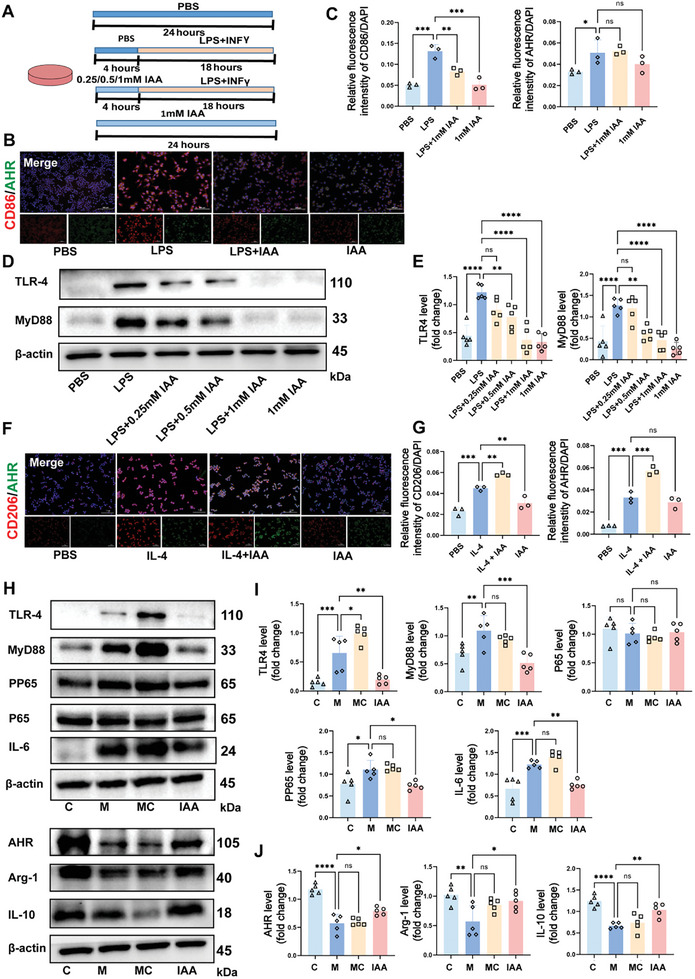
IAA regulates the M1/M2 macrophage balance to reduce inflammation. A) Experimental design: M1 polarisation was induced with IAA pretreatment for 4 h, followed by LPS and INFγ for 18 h. B) Representative image of CD86 and AHR immunofluorescence staining (Scale bar: 100 µm). C) Relative fluorescence intensity of CD86 and AHR (*n* = 3 independent experiments). D) Representative images of TLR4 and MyD88 western blots. E) Relative protein expression of TLR4 and MyD88 (*n* = 5 independent experiments). F) Representative image of CD206 and AHR immunofluorescence staining represented the image (Scale bar: 100 µm). G) Relative fluorescence intensity of CD206 and AHR (*n* = 3 independent experiments). H) Representative TLR4, MyD88, NF‐Κb(p65), P‐NF‐Κb (PP65), IL‐6, AHR, Arg‐1 and IL‐10 western blots. I, Relative protein expression of TLR4, MyD88, NF‐Κb(p65), P‐NF‐Κb (PP65), IL‐6, *n* = 5 mice/group. J) Relative protein expression of AHR, Arg‐1, and IL‐10, *n* = 5. Summary data are presented as the mean ± SEM. Statistical significance was determined using one‐way analysis of variance (ANOVA) followed by Dunnett's multiple comparison test for group comparisons. ns, not significant, *p < 0.05, **p < 0.01, ***p < 0.001, ****p < 0.0001. Abbreviations: LPS, lipopolysaccharide. INFγ, interferon gamma. IL‐4, inteleukin‐4. IAA, Indole‐3‐acetic acid. AHR, aryl hydrocarbon receptor. TLR4, Toll‐like receptor 4. MyD88, Myeloid differentiation factor. IL‐6, interleukin 6. Arg‐1, arginase‐1.

In animal models, an HFD disrupts the balance of macrophage M1/M2 polarisation, a process that IAA has been shown to restore, thereby mitigating AS through its effects on macrophages. To investigate this mechanism, we induced M1 polarisation in RAW264.7 cells by using classical stimulants (**Figure** **6**A). Immunofluorescence staining of CD86 and AHR revealed that IAA inhibited LPS + interferon‐gamma (INFγ)‐induced M1 polarisation, independently of AHR activation (Figure 6B,C). Western blotting further demonstrated a dose‐dependent decrease in the expression of TLR4 and MyD88, key inflammatory proteins, as the IAA concentration increased (Figure 6D,E). These results suggest that IAA reduces M1 polarisation by modulating the TLR4/MyD88 signaling pathway. To explore the impact of IAA on M2 polarisation, we induced M2 macrophages using IL‐4(Figure S6B, Supporting Information). Immunofluorescence staining for CD206 and AHR showed that IAA promoted M2 polarisation in an AHR‐dependent manner (Figure 6F,G), indicating that IAA has a dual role in regulating macrophage polarisation. In addition, when RAW264.7 cells were exposed to palmitic acid combined with LPS and INFγ to simulate a high‐fat condition, IAA reduced lipid uptake in macrophages (Figure S6C,D, Supporting Information), further emphasizing its role in mitigating inflammatory and metabolic disturbances associated with AS.

To elucidate the mechanism by which IAA affects AS, we treated the mouse aorta with IAA. Western blotting confirmed activation of the TLR4/MyD88/NF‐κB pathway and the inflammatory factor IL‐6, both of which were reversed by IAA treatment. Moreover, IAA enhanced the expression of anti‐inflammatory factors, including Arg‐1 and IL‐10, potentially through AHR receptor activation (Figure 6H,I). Finally, we intervened on IAA‐induced M2 macrophages with CH223191, an inhibitor of AHR, and found that the induction was reversed (Figure S6E,F, Supporting Information). Collectively, these findings highlight IAA as a crucial microbial metabolite that can attenuate the progression of AS by modulating macrophage polarisation and reducing inflammation.

## 3 Discussion

Modulation of the gut microbiota in the management of AS has emerged as a promising therapeutic strategy. While studies have highlighted the pivotal role of the gut microbiota in AS development, the specific microbial communities involved and their underlying mechanisms remain poorly understood. Furthermore, the complex interplay between diet, gut microbiota, and host metabolism remains inadequately defined, limiting the identification of microbial and metabolic targets for effective AS therapy.^[^ ^19^ ^]^ Therefore, the identification of key microbial strains and metabolites is crucial for the development of novel therapeutic approaches for AS.

In this study, we investigated the relationship between the gut microbiota and AS, demonstrating for the first time that the second‐generation probiotic *B. ovatus* can enhance lipid metabolism and reduce inflammation. These findings provide a theoretical foundation for future therapeutic applications of this strain in AS and for the development of related interventions. We characterised the fecal microbiota composition of patients with ASCVD and volunteers, identifying *B. ovatus* as a key differential bacterium. To control for confounding factors, we included volunteers with risk factors for coronary heart disease such as hypertension, diabetes, or dyslipidemia. Our analysis revealed that patients with ASCVD exhibited significantly lower α‐diversity compared with the volunteers, with notable differences in β‐diversity. The gut microbiota of the volunteers was enriched in beneficial, metabolically active bacteria. Species‐level compositional analysis revealed that *B. ovatus* was significantly more abundant in the volunteers, particularly within the genus *Bacteroides*. Correlation analyses further indicated a negative association between *B. ovatus* abundance and key metabolic indicators, including the TC and TG levels. Recent studies in HFD‐fed mice have shown that *B. ovatus* colonization reduces insulin resistance, improves intestinal barrier integrity, and decreases systemic inflammation.^[^ ^20^ ^]^ Additionally, Ihekweazu et al.^[^ ^21^ ^]^ demonstrated that *B. ovatus* promotes IL‐22 production and alleviates trinitrobenzene sulfonate‐induced colonic inflammation. Another study showed that *B. ovatus* colonization increased the abundance of SCFAs and neurotransmitters in the intestine, thereby ameliorating neurological inflammation.^[^ ^22^ ^]^ Collectively, these findings suggest that *B. ovatus* may play a protective role in the pathogenesis of ASCVD, and its modulation of the gut microbiota may offer therapeutic benefits.

To further elucidate the role of gut microbiota in AS development and to assess whether these effects can be replicated in animal models, we performed FMT in HFD‐fed *Apoe* ^−/−^ mice using feces from patients with ASCVD and volunteers with risk factors for coronary heart disease. The results demonstrated that FMT from volunteers significantly reduced systemic inflammation, modulated lipid metabolism, and decreased plaque formation and inflammatory markers in the aortic wall. Transplants from volunteers also mitigated hepatic fat deposition and liver damage. Mechanistically, FMT from volunteers enhanced the expression of tight junction proteins, including occludin, claudin‐1, and ZO‐1, thereby restoring intestinal barrier integrity (Figure 2o). These findings are consistent with previous studies indicating that IAA, a bioactive metabolite produced by *B. ovatus*, activates the AHR in the intestinal epithelium, leading to increased expression of barrier genes such as ZO‐1 and occludin‐1 and a reduction in circulating LPS levels.^[^ ^20^ ^]^ Additionally, the enrichment of beneficial bacteria, including *Lactobacillus* and *Rhodococcus spp*. has been linked to increased production of tryptophan metabolites that promote AHR activation in nuclear fractions. Activation of AHR induces the expression of IL‐22 and Glucagon‐Like Peptide‐1 (GLP‐1), further strengthening intestinal barrier integrity.^[^ ^23^ ^]^ These findings support our data, demonstrating that *B. ovatus* in the feces of volunteers produces IAA, which triggers a cascade of responses that restore intestinal barrier function and reduce the translocation of harmful substances such as LPS into the bloodstream.

To further investigate the therapeutic effects of the differential bacterium *B. ovatus* on AS, we conducted targeted interventions by treating *Apoe* ^−/−^ mice with isolated *B. ovatus*. Notably, *B. ovatus* effectively modulated lipid metabolism, reduced inflammatory factors, and diminished plaque accumulation in the aortic wall. Bile acids are crucial regulators of various metabolic processes, including energy metabolism, cholesterol homeostasis, and inflammation. Li et al.^[^ ^24^ ^]^ reported a strong association between *B. ovatus* and alterations in bile acid profiles, including increases in glycodeoxycholic acid (GDCA), β‐Chenodeoxycholic acid (CDCA), and DCA. Consistently, our study revealed that *B. ovatus* elevated levels of hyodeoxycholic acid (HDCA) (Figure 3h), which were inversely correlated with the TC levels. HDCA has been shown to improve metabolic disorders in various mouse models by inhibiting intestinal FXR activity and upregulating hepatic oxysterol 7a‐hydroxylase (Cyp7b1), suggesting a shift in bile acid biosynthesis from the classical to an alternative pathway.^[^ ^25^ ^]^ In contrast, inhibition of intestinal FXR‐fibroblast growth factor 15 (FGF15) signaling has been shown to increase the expression of enzymes involved in selective bile acid synthesis, activation of hepatic FXR, and enhanced hepatic lipolysis.^[^ ^26^ ^]^ Furthermore, antagonism of intestinal FXR signaling improves obesity‐related metabolic dysfunction by modulating ceramide‐mediated gut‐hepatic pathways.^[^ ^27^ ^]^ Taken together, our findings provide new insights into how BSH produced by *B. ovatus* enhances lipid homeostasis through bile acid metabolism, inhibition of intestinal FXR and NPC1L1 signaling, decreased enterohepatic circulation, and activation of hepatic FXR and alternative bile acid pathways (Figure 3j), thereby alleviating AS. However, the exact mechanism by which *B. ovatus* modulates inflammation remains unclear, as changes in cholesterol levels did not correlate with improvements in the inflammatory profile.

Chronic inflammation is a key factor in the progression of AS, persisting even after significant cholesterol reduction. Microbial metabolites play an essential role in host metabolism and human health;^[^ ^28^ ^]^ however, their involvement in AS pathogenesis, particularly those derived from amino acids, remains underexplored. Recent studies have demonstrated that *B. ovatus* produces IAA, which activates the AHR and effectively mitigates HFD‐induced metabolic syndrome, hepatic steatosis, and associated inflammatory responses.^[^ ^29^ ^]^ Therefore, we proposed a new scientific hypothesis: IAA also improves inflammation in the aortic wall. To test this, we performed a tryptophan assay on fecal samples and confirmed that *B. ovatus* could indeed produce IAA in *Apoe* ^−/−^ mice (Figure S4n, Supporting Information). Furthermore, both fecal (Figure S4m, Supporting Information) and serum (Figure 3m) IAA levels were significantly elevated following delivery of *B. ovatus* via oral gavage, supporting our hypothesis. Notably, the serum IAA levels were significantly higher in the NP group compared with the ASCVD group, further validating the potential role of IAA in AS (Figure 3n). This compelling evidence strongly suggests that *B. ovatus* is capable of producing IAA, which may play an important role in modulating inflammation and influencing the progression of AS. Additionally, *Lactobacillus spp*. Produces IAA, has been reported to attenuate inflammatory responses in rheumatoid arthritis by modulating NF‐κB and Mitogen‐Activated Protein Kinase (MAPK) phosphorylation through AHR activation.^[^ ^30^ ^]^ Ji et al.^[^ ^31^ ^]^ demonstrated that IAA reduces LPS‐induced inflammatory responses and free radical production in RAW264.7 macrophages by inducing Heme Oxygenase 1 (HO‐1) expression and directly neutralizing free radicals. Our mouse experiments showed that IAA inhibits inflammation and chemokine production, restores M1/M2 macrophage polarisation in atherosclerotic plaques, and reduces lipid deposition in both the liver and aortic wall (Figure 4h).

At the molecular level, TLR4 is overexpressed in atherosclerotic lesions and plays a critical role in regulating macrophage polarisation, making this pathway a potential therapeutic target. LPS binding to TLR4 drives macrophage polarisation toward the M1 phenotype, promoting the expression of pro‐inflammatory markers such as TNF‐α, IL‐1β, IL‐6, and Monocyte Chemotactic Protein 1 (MCP‐1), all of which are reversed by IAA. RNA‐seq of aortic tissues revealed significant changes in the expression of macrophage‐related genes among the top 30 upregulated DEGs. GO enrichment analysis indicated that monocytes, the precursors of macrophages in atherosclerotic plaques, were associated with enhanced migration and differentiation pathways, as well as increased macrophage activation. KEGG pathway analysis further revealed robust activation of the NF‐κB pathway, emphasizing the central role of inflammation in the pathogenesis of AS. Recent mechanistic studies suggest that IAA alleviates hepatic steatosis in an AHR‐dependent manner in hepatocytes; however, its anti‐inflammatory effects in macrophages appear to be mediated through Adenosine 5′‐Monophosphate (AMP)‐Activated Protein Kinase (AMPK) rather than AHR.^[^ ^32^ ^]^ In contrast, other studies have shown that cigarette smoke extract reduces IL‐22 production in RAW264.7 macrophages, while IAA restores IL‐22 levels in CSE‐stimulated macrophages and inhibits TNF‐α, with AHR inhibition attenuating these effects.^[^ ^33^ ^]^ Intriguingly, our results demonstrate that IAA inhibits TLR4/MyD88/NF‐κB pathway activation, reducing M1 macrophage polarisation through a mechanism independent of AHR. Furthermore, for the first time, we have shown that IAA promotes M2 macrophage polarisation and enhances the expression of anti‐inflammatory markers such as Arg‐1 and IL‐10, a key cytokine in inflammation resolution, in an AHR‐dependent manner (Figure 5h,i). Collectively, these findings suggest that IAA modulates the balance between pro‐inflammatory and anti‐inflammatory factors, thereby mitigating AS.

Our study has several limitations. First, we did not conduct clinical trials to evaluate the potential health effects of *B. ovatus* or IAA in human participants, as such interventions could carry risks. Second, the precise mechanisms by which IAA modulates the gut microbiota remain unclear, particularly in terms of its influence on interbacterial signaling molecules that regulate microbial communities. Finally, while we explored the roles of AHR in macrophage polarisation, the distinct functions of AHR in M1 and M2 macrophages require further investigation and will be the focus of future studies.

## 4 Conclusion

In conclusion, our findings demonstrate, for the first time, that the second‐generation probiotic *B. ovatus* ameliorates AS. This strain restores the balance of M1/M2 macrophage polarisation by enhancing intestinal barrier function, improving bile acid and lipid metabolism, and, importantly, producing IAA. This metabolite inhibits the TLR4/MyD88/NF‐κB pathway in M1 macrophages while promoting M2 macrophage polarisation to reduce inflammation. Our study clarifies the relationship between gut microbes and AS, enhancing our understanding of the diet‐gut‐liver‐aorta‐macrophage axis in AS pathogenesis. These results provide a theoretical foundation for the future therapeutic application of *B. ovatus* in AS and the development of related formulations.

## 5 Experimental Section

### Human Subjects and Sample Collection

The clinical cohort was recruited from the Second Affiliated Hospital of Nanchang University and consisted of 60 participants. The inclusion criteria for the ASCVD group included patients with stable angina (encompassing asymptomatic myocardial ischemia, stable angina pectoris, and ischemic cardiomyopathy) or a history of myocardial infarction; a preserved left ventricular ejection fraction (>40%); and evidence of coronary artery disease on coronary angiography, including single stenosis of ≥50% or multiple stenoses. A history of coronary artery disease was defined by documented angina pectoris, abnormalities on a 24‐h ambulatory electrocardiogram (indicating old myocardial infarction), or abnormal chest radiographs. The control (NP) group included individuals with risk factors for coronary heart disease (e.g., hypertension, diabetes mellitus, or dyslipidaemia) but without a diagnosis of ASCVD. The NP group was matched to the ASCVD group in terms of age and sex. The exclusion criteria were: 1) acute coronary syndromes, (e.g., non‐ST‐elevation acute myocardial infarction, unstable angina, and ST‐elevation myocardial infarction [plaque rupture]); 2) systemic diseases, such as liver disease, renal disease (serum creatinine level >2.0 mg dL^−1^), or malignancies; and 3) patients receiving antibiotic therapy. The participants completed detailed questionnaires on their medical history, lifestyle, and dietary habits and provided stool and serum samples. Disposable stool sampling kits, accompanied by detailed instructions, were provided for sample collection. Stool samples were preserved in 30% glycerol immediately after collection and stored at ‐80 °C within 2 h to maintain their integrity. All stool samples underwent full‐length 16S rRNA gene high‐throughput sequencing for microbiome analysis. Diabetes was defined as a glycated hemoglobin level >6.5%, the use of oral hypoglycaemic agents, or receiving insulin therapy. Hypertension was defined as blood pressure ≥140/90 mmHg or the use of antihypertensive drugs. Dyslipidaemia was defined as LDL‐c level >140 mg dL^−1^, TG >150 mg dL^−1^, or the use of anti‐hypertensive drugs.

### Mice and Treatments

Healthy 6‐8‐week‐old male *Apoe* ^−/−^ mice and wild‐type (C57BL/6J) mice were housed under a 12‐h photoperiod with ad libitum access to water and standard chow in an SPF animal facility. The mice were acclimatized for 1 week before the start of the experiments. *Apoe* ^−/−^ mice were fed a Western diet (HFD) to induce AS. Only male mice were used to eliminate the potential confounding effects of oestrogen, which is known to protect against AS in females. Experiment 1. This experiment aimed to assess the effects of FMT from the ASCVD and NP groups on AS progression. The mice body weights were monitored weekly, and fecal samples were collected at the end of the study. Blood samples were obtained post‐euthanasia, allowed to clot at room temperature for 2 h, and then centrifuged at 3000 rpm for 15 min at 4 °C. The supernatant was stored at −80 °C for subsequent analyses. The aorta, colon, liver, and spleen were isolated, photographed, and processed for further analysis. The heart, colon, liver, aortic root, and aortic arch were fixed in 4% paraformaldehyde for morphological and immunohistochemical evaluations or stored at −80 °C for molecular biology assays. Experiment 2. In this experiment, *B. ovatus* was isolated, and its functional impact on AS was examined. This bacterium was cultured anaerobically on blood agar plates (Brain Heart Infusion [BHI] medium containing 5% sheep blood and trypsin) at 37 °C for 48–72 h. The mice were administered 200 µL of bacterial culture every 2 days via oral gavage. Data were recorded, and blood and tissue samples were collected and processed as described in Experiment 1. Experiment 3. This experiment evaluated the effects of IAA, a tryptophan metabolite produced by *B. ovatus*, on AS. IAA was administered at a dose of 200 µL every 2 days via oral gavage. Data were recorded, and blood and tissue samples were collected as described in Experiment 1.

### Cell Culture and Stimulation

RAW264.7 cells, a mouse macrophage cell line, were seeded at a density of 2 × 10⁵ cells per well in 6‐well plates and cultured in Dulbecco's Modified Eagle's Medium (DMEM) supplemented with 10% foetal bovine serum, 100 µg mL^−1^ streptomycin, and 100 U/mL penicillin at 37 °C in a 5% CO₂ atmosphere. Experiment 1–M1 polarisation induction. M1 polarisation was induced with lipopolysaccharide (LPS) and interferon‐gamma (INFγ). The experimental groups included: control (phosphate‐buffered saline [PBS]); LPS (100 ng mL^−1^) + INFγ (10 ng mL^−1^); LPS + INFγ + IAA (0.25–1 mM); and IAA alone (1 mM). IAA was added 4 h before LPS stimulation, followed by incubation with LPS for 18 h. Experiment 2–M2 polarisation induction. M2 polarisation was induced using IL‐4, as described previously.^[^ ^34^ ^]^ The experimental groups included: control (PBS); IL‐4 (20 ng mL^−1^); IL‐4 + IAA (1 mM); and IAA alone (1 mM). IAA was added 4 h before LPS stimulation, followed by incubation with LPS for 18 h. Experiment 3 – induction of cellular hyperlipidemia. A cellular hyperlipidemia model was generated as described previously^[^ ^35^ ^]^ with palmitoleic acid (10 mM) combined with LPS (100 ng mL^−1^) and INFγ (10 ng mL^−1^). The experimental groups included: control (PBS); palmitoleic acid + LPS + INFγ; IAA + palmitoleic acid + LPS + INFγ; and IAA alone. Following a 4‐hour pretreatment with IAA, the cells were incubated with palmitoleic acid for 18 h; then, LPS and INFγ were added for an additional 6 hours. Experiment 4 –AHR inhibition. The AHR inhibitor CH223191 was used to investigate the AHR‐mediated effects of IAA. IL‐4 (20 ng mL^−1^) was used to induce M2 polarisation. The experimental groups included: control (PBS); IL‐4; IL‐4 + IAA (1 mM); and IL‐4 + IAA + CH223191 (10 µM). After a 4‐h pretreatment with IAA and CH223191, IL‐4 was added for an additional 18 hours.

### Fecal DNA Extraction, Data Analysis

Fecal samples were stored at −80 °C, and genomic DNA was extracted using the Genomic DNA Extraction Kit (Personalbio, Shanghai, China) following the manufacturer's protocol. The V1‐V9 regions of the 16S rRNA gene were amplified using universal primers under optimised thermal cycling conditions. The amplified DNA fragments were sequenced using a paired‐end approach on the Nova Seq 6000 (illumina, San Diego, California, USA). Raw sequencing data were processed for quality control using Vsearch (v2.13.4_linux_x86_64) and cutadapt (v2.3) to remove adapters, primers, and low‐quality sequences. Amplicon sequence variant (ASV) or representative operational taxonomic unit (OTU) sequences were obtained, and the length distribution of high‐quality sequences was assessed. Species annotation was conducted using the classify‐sklearn algorithm within the QIIME2 software package. Taxonomic classification was performed for each ASV or OTU sequence by using a pre‐trained naïve Bayes classifier, comparing sequences to reference databases such as Greengenes, NT, and UNITE. Taxonomic annotations were performed at multiple taxonomic levels (kingdom, phylum, class, order, family, genus, and species). Subsequent analyses included an evaluation of the species composition, alpha (α)‐diversity, and beta (β)‐diversity. Additionally, differential species analysis, marker species identification, association network construction, and functional predictions were conducted to provide insights into the microbial community structure and its potential functional implications.^[^ ^36^ ^]^

### Metabolomics

Metabolomics was performed using liquid chromatography‐tandem mass spectrometry (LC‐MS/MS) to quantitatively measure 65 bile acids and 31 tryptophan metabolites in fecal samples. A custom Metware Database (MWDB), developed by Wuhan Metware Biotechnology Co. (Wuhan, china), was used for data analysis. Quantitative MS data were acquired using triple quadrupole MS in the multiple reaction monitoring (MRM) mode by QTRAP 6500+ (SCIEX, Massachusetts, USA). The MultiQuant 3.0.3 software (SCIEX, Massachusetts, USA) was employed to process the MS data, using the retention time and peak shape information from standards to perform integral corrections for chromatographic peaks. The ratios of integral peak areas from the detected samples were used to calculate the metabolite concentrations by applying linear equations derived from standard curves. The acquired metabolomic data were subjected to comprehensive statistical analyses, including principal component analysis (PCA), orthogonal partial least squares discriminant analysis (OPLS‐DA), and cluster analysis. Differential metabolite screening was performed to identify significant changes in metabolite profiles.

### FMT

FMT was performed according to established protocols.^[^ ^37^ ^]^ Before transplantation, the mice underwent antibiotic treatment with a cocktail consisting of vancomycin (100 mg k^−1^g), neomycin sulfate (200 mg k^−1^g), metronidazole (200 mg k^−1^g), and ampicillin (200 mg k^−1^g) administered via oral gavage once daily for 1 week to deplete their native gut microbiota.^[^ ^38^ ^]^ The mice were monitored closely for signs of dehydration during antibiotic administration. Donor screening was based on predefined inclusion and exclusion criteria for patients with ASCVD, while the control volunteers were selected based on principal coordinate analysis (PCoA) of Bray – Curtis distances to ensure a significant compositional difference from the patients with ASCVD. Fresh fecal samples from patients with ASCVD and the volunteers were immediately mixed with five volumes of sterile saline, homogenized, and processed by filtration and centrifugation (100 *g* for 5 min at 4 °C). The resulting supernatant was suspended in 30% glycerol and stored at −80 °C until use. For FMT, the stored fecal preparations were thawed, centrifuged, resuspended in gelatine saline, and administered to recipient mice via oral gavage.

### Detection of Probiotic Properties

*B. ovatus* was activated overnight and cultured in BHI medium supplemented with 5% sheep blood and trypsin. The optical density (OD) at 600 nm was measured every 2 h until no further increase was observed, indicating that the bacteria had reached the stationary phase. A growth curve was generated, with time on the x‐axis and the OD values on the y‐axis. To assess the effect of bile salts, 0.1%–0.3% bovine bile salts were added to the BHI medium, and the culture was incubated until the stationary phase. A 10‐µL aliquot was plated on anaerobic blood agar plates to quantify the number of viable bacteria. For antibacterial activity, selected pathogenic bacteria were cultured to the stationary phase in appropriate liquid media and plated onto Luria‐Bertani (LB) agar plates. Oxford cups were placed on each plate, and 250 µL of the bacterial culture supernatant was added to the cup. After incubation for 8 hours, the inhibition zone diameter was measured to assess antibacterial activity. The antimicrobial susceptibility of *B. ovatus* to 10 common antimicrobials was tested using the Kirby‐Bauer (K‐B) disk diffusion method, following the Clinical and Laboratory Standards Institute (CLSI, 2018 edition) guideline. Briefly, *B. ovatus* cultures were spread on anaerobic blood agar plates, and antibiotic‐impregnated disks were placed on the agar surface. After incubation for a 24‐h, the inhibition zone diameters were measured. For the cell adhesion assay, HT29 cells were cultured in DMEM/F‐12 medium supplemented with 5% foetal bovine serum, 100 U mL^−1^ penicillin, and 100 µg mL^−1^ streptomycin. When the cells reached 95% confluence, they were seeded onto 6‐well plates containing glass coverslips at a density of 5 × 10⁶ cells per well and allowed to adhere overnight. *B. ovatus* was added to the plates (1 × 10⁷ colony‐forming units [CFU]) and co‐incubated with HT29 cells for 2 h at 37 °C. After incubation, the cells were fixed with formaldehyde, stained, and examined under a light microscope (Nikon) to evaluate bacterial adhesion.

### Plasma Analysis

Serum samples were stored at −80 °C until analysis of the cytokine and lipid profile. TG, TC, and LDL‐c were measured using commercial assay kits (Jiangsu Edison Biotechnology Co., Ltd., Supporting Information), according to the manufacturer's instructions. Inflammatory markers, including LPS, TNF‐α, IL‐6, and IL‐1β, were also quantified. Target analytes such as IAA in both serum and fecal samples, were detected using commercially available kits.

### Assessment of Atherosclerotic Plaques

Atherosclerotic lesions were assessed as described previously.^[^ ^39^ ^]^ The entire aorta was excised following adequate fixation, washed with saline or PBS to remove residual fixative, and then stained with oil red O for 2 h at room temperature. Adjacent tissues were carefully excised, and the aorta was opened longitudinally for detailed analysis. For the evaluation of atherosclerotic lesions in the aortic sinus, the proximal aorta attached to the heart was harvested and fixed in 4% paraformaldehyde, while the remaining sections were rapidly frozen in liquid nitrogen for subsequent qPCR or western blotting. The formaldehyde‐fixed hearts were transferred to 25% sucrose in PBS for over 48 hours until they had completed dehydrate (i.e., sunk to the bottom of the solution). To prepare for cryosectioning, the heart was removed, excess moisture was blotted with filter paper, and the apical two‐thirds of the heart was excised perpendicular to the long axis using a blade. The remaining heart tissue was placed cut‐side down in a frozen section embedding mold, submerged in optimal cutting temperature compound, and frozen using a cryostat. The frozen tissue blocks were wrapped in aluminum foil and stored at −80 °C for further histological and molecular analyses. Masson's trichrome staining was used to assess the collagen content and to examine the aortic fibrous cap, enabling differentiation between collagen fibers, myofibres, and other cellular structures. To measure the thickness of the fibrous cap, the largest necrotic core in each mouse section was selected, and the area between the outer edge of the fibrous cap and the boundary of the necrotic core was averaged. Morphometric measurements of collagen content and fibrous cap thickness were performed using the ImageJ (National Institutes of Health, Bethesda, MD, USA), enabling precise quantification of lesion area.

### Histology, Immunostaining and Immunofluorescence

Aortic, liver, and colon tissue specimens were embedded in paraffin, sectioned into 4‐µm thick slices, and stained with hematoxylin and eosin (HE) to assess tissue alterations and morphological features under a light microscope (Eclipse 80i, Nikon, Tokyo, Japan) using the NIS‐Elements 3.2 software. Liver sections were independently evaluated for the Non‐Alcoholic Fatty Liver Disease (NAFLD) Activity Score (NAS, liver Score) by two pathologists, with discrepancies resolved by a third physician. The NAS assessed hepatocellular steatosis (0–3), score 1 (0%–33%), score 2 (33%–66%), and score 3 (66%–100%); hepatocellular ballooning (0–2), score 0 (no ballooning), score 1 (mild ballooning), and score 2 (severe ballooning); and intrahepatic lobular inflammation (0–3), score 1 (0–1 necrotic foci), score 2 (2–4 necrotic foci), and score 3 (>4 necrotic foci).^[^ ^40^ ^]^ Colonic tissue was assessed by measuring crypt depth and the number of AB‐PAS (Alcian Blue‐Periodic Acid Schiff)–stained goblet cells.^[^ ^41^ ^]^ Immunohistochemical staining was performed to assess the expression of aortic chemokines, colonic barrier proteins, and cholesterol transporters. The sections were subjected to antigen retrieval in citric acid (pH 6.0), followed by incubation with a buffer containing 10% donkey serum and 5% bovine serum albumin (BSA) for 1 h to block nonspecific protein binding. The sections were incubated with the appropriate primary antibody overnight at 4 °C, washed with PBS, and then incubated with horseradish peroxidase (HRP)‐conjugated secondary antibody for 50 min at 37 °C. After staining with hematoxylin, dehydrating, and sealing with neutral adhesive, immunopositive cells were visualized using a light microscope (Nikon). For immunofluorescence double staining, Cryosections were prepared and treated with 0.5% Triton X‐100 for 20 min and then washed with PBS. The sections were incubated with 3% BSA for 1 h to block non‐specific protein binding, and then incubated with the appropriate primary antibody overnight at 4 °C. After washing, the sections were incubated with FITC (green) or Cy3 (red) conjugated Goat Anti‐Rabbit IgG secondary antibody for 2 h in the dark, followed by a final PBS wash. An anti‐fluorescence quenching sealer was applied, and the samples were mounted in glycerol for analysis.

### RNA Extraction and Real‐Time qPCR

Total RNA from fecal samples was extracted using the QIAamp Fecal DNA Rapid Purification Mini Kit, following the manufacturer's instructions. RNA from tissue samples was extracted using TRIzol reagent. The RNA concentration was measured using a NanoDrop 2000 spectrophotometer (Thermo Fisher Scientific, Wilmington, DE, USA). Reverse transcription was performed with the PrimeScript RT Reagent Kit (with gDNA Eraser) to synthesize complementary DNA (cDNA). QPCR was conducted using TB Green Premix Ex Taq II on a ViiA 7 Real‐Time PCR System (Applied Biosystems, Carlsbad, California, USA). The relative messenger RNA (mRNA) expression levels of target genes were calculated using the 2^−ΔΔCt^ method.

### RNA‐Seq and Transcriptomic Analysis

Genome‐wide gene expression analysis was performed on aortic tissues from mice fed a normal diet (*n* = 3) and *Apoe* ^−/−^ mice fed an HFD (*n* = 3). RNA extraction, quality control, library preparation, and sequencing were conducted by Shanghai WeiHuan Biotechnology Co., Ltd. (https://www.apexbio.cn/). DEGs were identified based on *p* < 0.05 and a fold change ≥1.5. Differential expression analysis was carried out using DESeq2 for samples with biological replicates or edgeR for non‐replicated samples, followed by heatmap visualization. GO and KEGG enrichment analyses were performed using a hypergeometric distribution algorithm to identify significantly enriched functional categories. Gene Set Enrichment Analysis (GSEA) was conducted using GSEA software (http://www.broad.mit.edu/GSEA) to compare gene expression profiles between vector‐ and IAA‐treated mice.

### Western Blotting

Proteins were extracted from the gut, liver, aortic tissue, and RAW264.7 cells using radioimmunoprecipitation assay (RIPA) lysis buffer supplemented with protease and phosphatase inhibitors. The protein concentration was determined using the BCA Protein Quantification Kit. Equal amounts of protein were separated by 8%–12% sodium dodecyl sulfate‐polyacrylamide gel electrophoresis (SDS‐PAGE) and transferred onto 0.2–0.45 µm polyvinylidene difluoride (PVDF) membranes. After incubation with 5% nonfat milk for 2 h at room temperature to block non‐specific protein binding, the membranes were incubated with the appropriate primary antibody overnight at 4 °C. Following three washes with 0.3% Tris‐buffered saline with Tween‐20 (TBST), the membranes were incubated with the appropriate secondary antibody for 2 h at room temperature. The membranes were exposed to an ultrasensitive chemiluminescence substrate (Super ECL) for 1 min and analyzed using a fully automated gel imaging system (Tanon 5200, shanghai, China). Protein levels were quantified with the ImageJ software.

### Statistical Analyses

The number of animals used in each study is provided in the Figure legend, with *n* representing the number of biological replicates. Statistical analyses were performed using GraphPad Prism version 9 (GraphPad Software, San Diego, CA, USA) and SPSS Statistics version 26 (IBM Corp., Armonk, NY, USA). For clinical cohort analysis, the normality of variable distributions was assessed using the Shapiro‐Wilk test. Normally distributed continuous variables are reported as the mean ± the standard error of the mean (SEM) or the standard deviation (SD), while non‐normally distributed variables are expressed as the median (interquartile range). Categorical variables are reported as counts and percentages. Differences between groups were assessed using the independent samples chi‐square (χ^2^) test for categorical variables, the independent samples t‐test for normally distributed continuous variables, and the Mann‐Whitney U test for non‐normally distributed continuous variables. Correlations between clinical indicators and primary changing microorganisms were assessed using Spearman's correlation analysis, as described previously, using the corrplot package in R version 4.4.0. Statistical significance was determined using one‐way analysis of variance (ANOVA) followed by Dunnett's multiple comparison test for comparisons, and unpaired t‐test for two‐group comparisons. A two‐sided *p*‐value of <0.05 was considered to indicate a statistically significant difference.

### Ethics Approval and Consent to Participate

All animal experiments were approved by the Laboratory Animal Welfare Ethics Committee of Nanchang University (Approval No. NCULAE‐20221031130). The study was approved by the Ethics Committee of the Second Affiliated Hospital of Nanchang University (Approval No. 2 023 061), and written informed consent was obtained from all participants.

## Conflict of Interest

The authors declare no conflict of interest.

## Author Contributions

Y.‐H.T. and T.‐T.C. contributed equally to this work. W.L., Y.‐H.T., and T.‐T.C. designed the experiments. W.L. and J.‐Y.W. conducted the experiments. H.Y. and C.‐C.L. examined and interpreted the data. W.L. and W.‐Q.L. drafted the paper, and Y.‐H.T. and T.‐T.C. revised the manuscript. All authors read and approved the final manuscript.

## Supporting information



Supporting Information

Supporting Information

Supporting Information

## Data Availability

The raw 16S rRNA sequencing data generated for the human and murine experiments and aortic RNA‐seq data have been deposited in the NCBI Sequence Read Archive under the accession numbers PRJNA1163052, PRJNA1163334 and PRJNA1189690.
