# Supplementary material for: The Metabolite Indole‐3‐Acetic Acid of Bacteroides Ovatus Improves Atherosclerosis by Restoring the Polarisation Balance of M1/M2 Macrophages and Inhibiting Inflammation
Source: Adv Sci (Weinh). 2025 Jan 22;12(11):2413010. doi: 10.1002/advs.202413010 (PMC11924036; doi:10.1002/advs.202413010)
Supplement: Supplementary file 1 — Supporting Information [file ADVS-12-2413010-s001.docx]

**Table S1. Primer sequences used in real-time quantitative RT-PCR**

| Gene | Species | Forward | Reverse |
| --- | --- | --- | --- |
| *Cyp27a1* | mouse | TCCCAGTGTCTTTCCTGAGC | CACAGAGCCGAATGGATGTA |
| *Cyp7a1* | mouse | GTCCGGATATTCAAGGATGC | GGGAATGCCATTTACTTGGA |
| *Cyp7b1* | mouse | TGAGGTTCTGAGGCTGTGC | TGGAGGAAAGAGGGCTACAA |
| *FXR* | mouse | CCCCTGCTTGATGTGCTAC | CGTGGTGATGGTTGAATGTC |
| *FGF15* | mouse | TGTTTCACCGCTCCTTCTTT | TCTACATCCTCCACCATCCTG |
| *TNF-α* | mouse | CGGGCAGGTCTACTTTGGAG | CAGGTCACTGTCCCAGCATC |
| *IL-6* | mouse | CCAAGAGGTGAGTGCTTCCC | CTGTTGTTCAGACTCTCTCCCT |
| *IL-1β* | mouse | TTCGTGAATGAGCAGACAGC | GGTTTCTTGTGACCCTGAGC |
| *AHR* | mouse | ACATCACCTACGCCAGTCGC | TCTATGCCGCTTGGAAGGAT |
| *MCP-1* | mouse | CTTCTGGGCCTGCTGTTCA | CCAGCCTACTCATTGGGATCA |
| *ABCA1* | mouse | GGACTTGCCTTGTTCCGAGAG | GCTGCCACATAACTGATAGCGA |
| *β-Actin* | mouse | GATGTATGAAGGCTTTGGTC | TGTGCACTTTTATTGGTCTC |
| *BO* | mouse | TGCAAACTRAAGATGGC | CAAACTAATGGAACGCATC |
| *BL* | mouse | CACCGCTACACATGGAG | AGCAGTAGGGAATCTTCCA |
| *BI* | mouse | TCGCGTC(C/T)GGTGTGAAAG | CCACATCCAGC(A/G)TCCAC |
| *BA* | mouse | GGTGTCGGCTTAAGTGCCAT | CGGA(C/T)GTAAGGGCCGTGC |
| *16s* | mouse | ACTCCTACGGGAGGCAGCAGT | TATTACCGCGGCTGCTGGC |

*BO:Bacteroides ovatus; BL:Lactobacillus; BI:Bifidobacterium; BA:Bacteroides*.

**Table S2. Antibodies used in the article**

| Target antigen | Vendor | Catalog# | Concentration |
| --- | --- | --- | --- |
| anti-β-Actin antibody | Cell Signaling  Technology | 4967S | 1:1,000 |
| anti-Arg-1 antibody | Servicebio | GB11285 | 1:5,000 |
| anti-AHR antibody | Proteintech | 28727-1 | 1:5,000 |
| anti-FXR1 antibody | Proteintech | 13194-1 | 1:20,000 |
| anti-CYP27A1 antibody | Boster | bs-5049R | 1:2,000 |
| anti-TLR4 antibody | Proteintech | 66350-1 | 1:4,000 |
| anti-NF-κB p65 antibody | Proteintech | 10745-1 | 1:500 |
| anti-Phospho-NF-κB p65 antibody | Cell Signaling  Technology | 3033S | 1:1,000 |
| anti-MyD88 antibody | Cell Signaling  Technology | 4283S | 1:1,000 |
| anti-IL-6 antibody | Cell Signaling  Technology | 12912S | 1:1,000 |
| anti-IL-10 antibody | ABclonal | A12255 | 1:1,000 |
| anti-Claudin-1 antibody | Abcam | ab180158 | 1:2,000 |
| anti-Occludin antibody | Proteintech | 66378-1 | 1:5,000 |
| anti-NPC1L1 antibody | Boster | bs-8849R | 1:500 |
| anti-ICAM-1 antibody | Servicebio | GB11106 | 1:500 |
| anti-VCAM-1 antibody | Servicebio | GB113498 | 1:1,000 |
| anti-Occludin antibody | Servicebio | GB11140 | 1:500 |
| anti-ZO-1 antibody | Servicebio | GB111402 | 1:500 |
| anti-CD68 antibody | Servicebio | GB113109 | 1:100 |
| anti-CD206 antibody | Servicebio | GB113497 | 1:400 |
| anti- AHR antibody | Proteintech | 28727-1 | 1:500 |
| Anti-Mouse secondary antibody | Proteintech | SA00001-1 | 1:2,000 |
| Anti-Rabbit secondary antibody | Proteintech | SA00001-2 | 1:2,000 |
| FITC(green) conjugated Goat Anti-Rabbit IgG | Servicebio | GB22303 | 1:100 |
| Cy3(red) conjugated Goat Anti-Rabbit IgG | Servicebio | GB21303 | 1:100 |

**Table S3. Key Resources**

| Chemicals, Peptides, and Recombinant Proteins | | |
| --- | --- | --- |
| LPS | Sigma-Aldrich | Cat#L4391 |
| INFγ | Peprotech | Cat#315-05 |
| IL-4 | Peprotech | Cat#214-14 |
| Sodium palmitate | Sigma-Aldrich | Cat#SLCN9277 |
| Albumin, from bovine Fatty free | MeilunBio | Cat#MB0094-1 |
| indole-3-acetic acid | Sigma-Aldrich | Cat#I5148 |
| CH223191 | MedChemExpress | Cat#HY-12684 |
| TRIzol reagent | Takara | Cat#9109 |
| Dulbecco's Modified Eagle Medium(DMEM) | Gibco | Cat#C11965500BT |
| foetal bovine serum(FBS) | Gibco | Cat#16000-044 |
| Penicillin and Streptomycin | Beyotime Biotechnology | Cat#C0222 |
| optimal cutting temperature compound | Servicebio | Cat#G6059-110ML |
| radioimmunoprecipitation assay (RIPA) | Solarbio | Cat#R0020 |
| polyvinylidene difluoride (PVDF) | Merck | Cat#ISEQ00010 |
| Tween-20 | Solarbio | Cat#T8220 |
| ultrasensitive chemiluminescence substrate | UElandy | Cat#S6009M |
| Critical Commercial Assays | | |
| QIAamp Faecal DNA Rapid Purification Mini Kit | QIAGEN GmbH | Cat#51604 |
| PrimeScript™RT Reagent Kit with gDNA Eraser | Takara | Cat#RR047A |
| TB Green®Premix Ex Taq™ II | Takara | Cat#RR820A |
| Genomic DNA Extraction Kit | MP Biomedicals | Cat#116564384 |
| BCA Protein Quantification Kit | Thermo Fisher | Cat#23227 |
| TG assay kits | Edison Bio | Cat#W-ZF013 |
| TC assay kits | Edison Bio | Cat#W-ZF014 |
| LDL-C assay kits | Edison Bio | Cat#W-D012 |
| IL6 ELISA Kit | Boster | Cat#EK0411 |
| IL-1 beta ELISA Kit | Boster | Cat#EK0394 |
| TNF Alpha ELISA Kit | Boster | Cat#EK0527 |
| CD95/FAS ELISA Kit | Boster | Cat#EK0336 |
| IAA ELISA kits | Lunchangshuo Biotech | Cat#LCSJZF99908 |
| Experimental Models: Organisms/Strains | | |
| *Apoe^-/-^* | Huachuang Sino and Sipeifu Laboratory Animal Technology | N/A |
| C57BL/6J |  |  |
| Western diet | Adjusted Calories Diet: 40 kcal %, 1.25% cholesterol, and 0.5% sodium cholate | Cat#TD.88137 |
| Standard chow | 10 kcal % |  |
