# Supplementary material for: The Metabolite Indole‐3‐Acetic Acid of Bacteroides Ovatus Improves Atherosclerosis by Restoring the Polarisation Balance of M1/M2 Macrophages and Inhibiting Inflammation
Source: Adv Sci (Weinh). 2025 Jan 22;12(11):2413010. doi: 10.1002/advs.202413010 (PMC11924036; doi:10.1002/advs.202413010)
Supplement: Supplementary file 3 — Supporting Information [file ADVS-12-2413010-s002.docx]

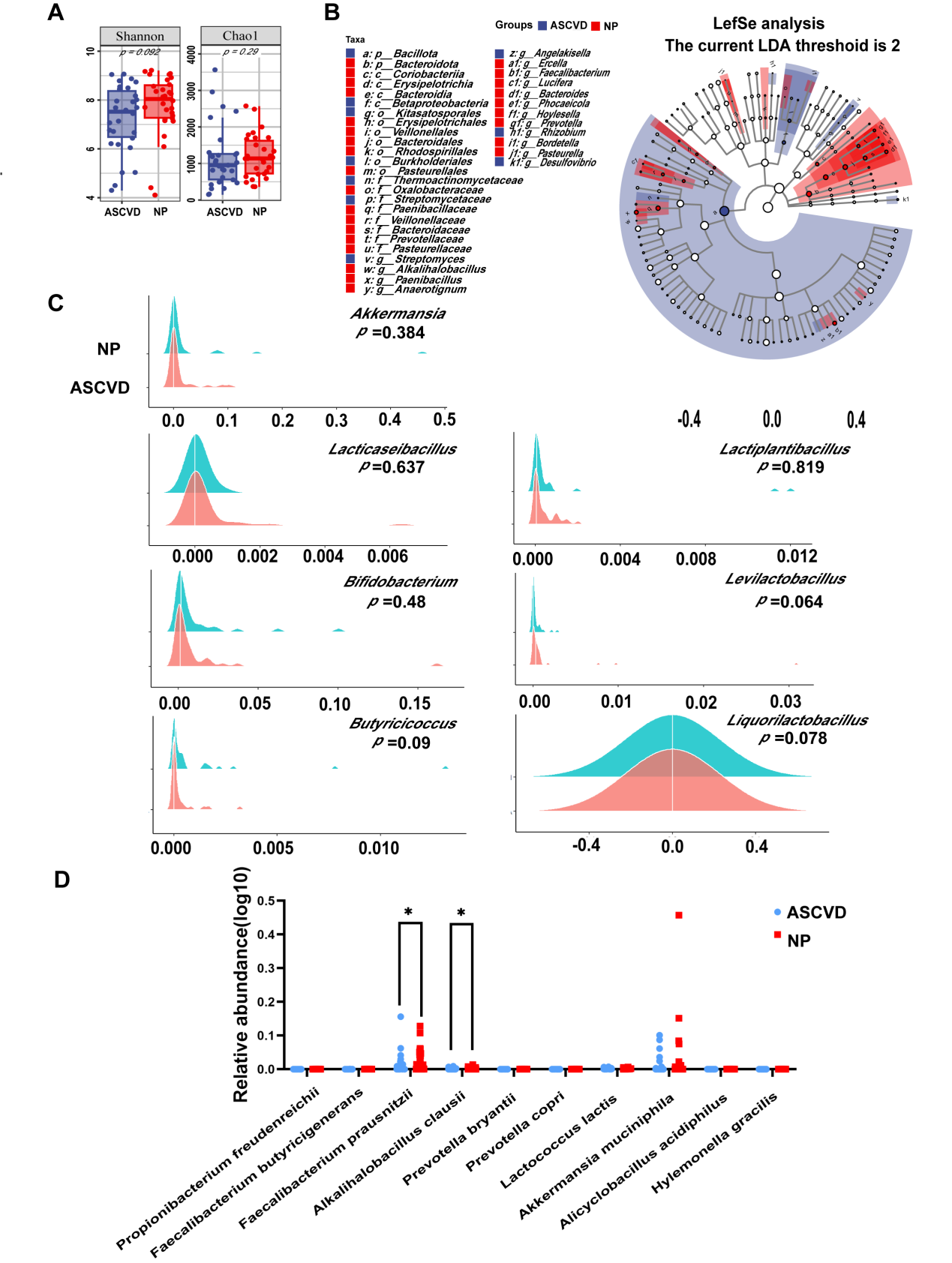


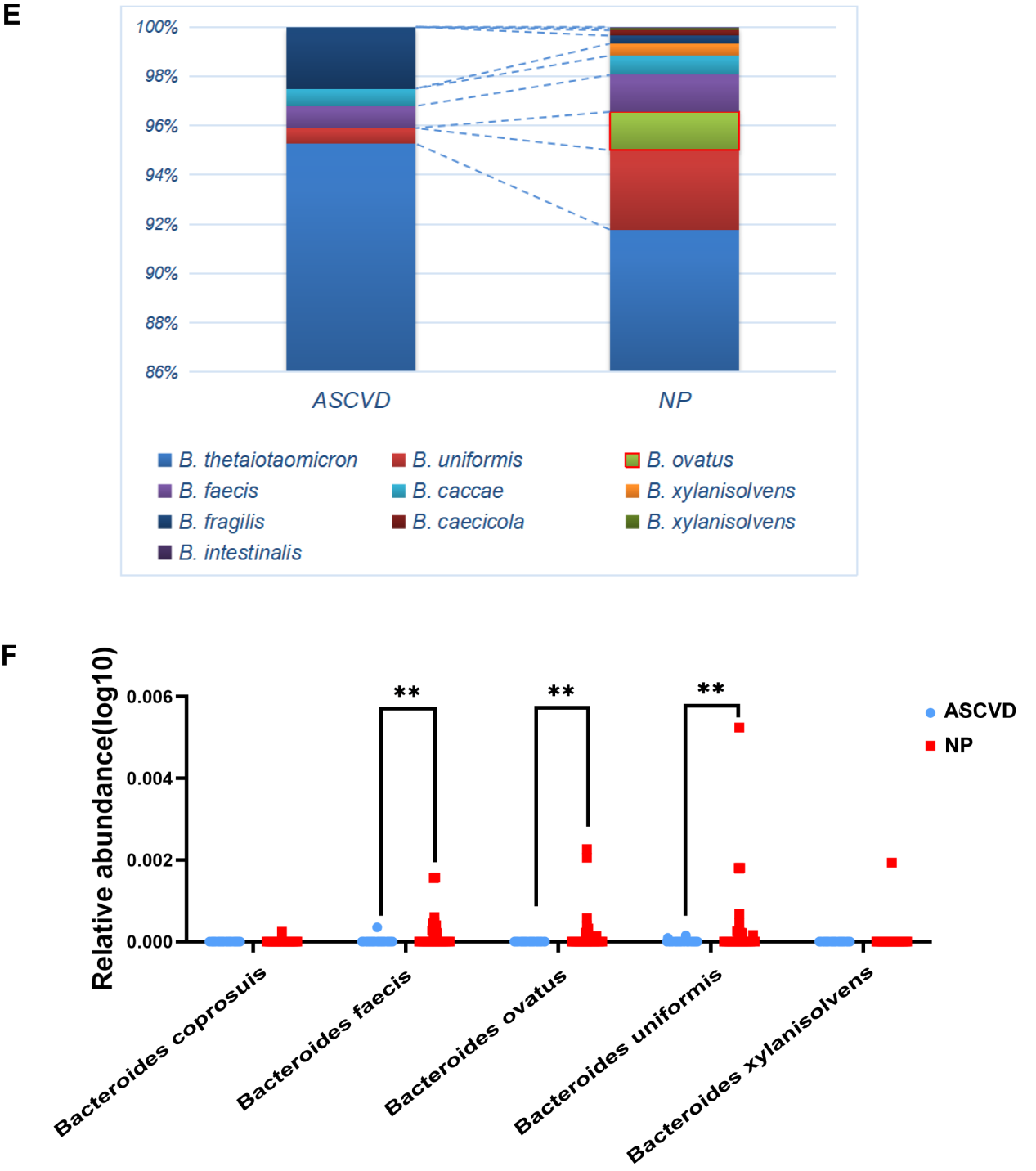


**Figure S1.** A) Alpha diversity of the microbial community. B) Classification branch graph generated by linear discriminant analysis effect size. The size of each circle is proportional to the abundance of the taxa. C) Map of the genus-level mountain ranges, n= 30. D) Relative abundance of the two groups of food catalogue strains, n= 30. E) *Bacteroides* species level percentage stacking histogram. F) Relative abundance of Bacteroides at the species level, n= 30. Summary data are presented as the mean ± SEM. Statistical significance was determined by the Mann-Whitney U test (C, D, and F). **P* <0.05, ***P* < 0.01. LDA, Linear discriminant analysis.


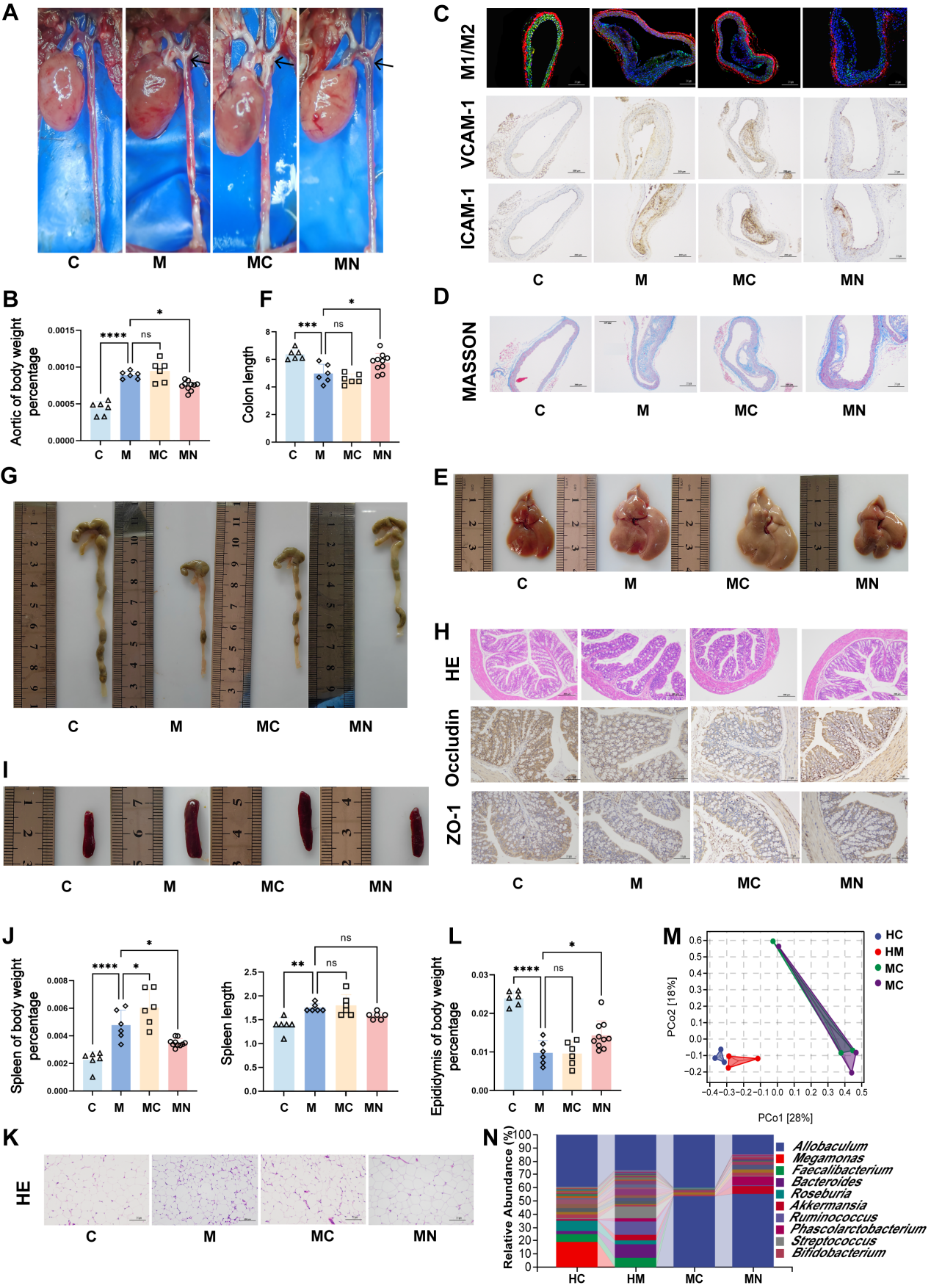


**Figure** **S2.** A) Gross anatomy of mouse aorta. B) Aortic weight, n=6-10 mice/group. **C**, Representative images of VCAM-1 and ICAM-1 immunofluorescence staining in the aorta (Scale bar: 200 μm). D) Representative image of aortic Masson’s trichrome staining (Scale bar: 200 μm). E) Gross anatomy of the liver. F) Colon length quantification, n=6-10. G) Gross anatomy of the colon. H) Representative images of HE (Scale bar: 200 μm), and ZO-1, occluding (Scale bar: 100 μm) staining in the colon. I) Gross anatomy of the spleen. J) Spleen weight (n=6-10) and length (n=6). K) Representative image of HE staining of the epididymis (Scale bar: 100 μm). L) Epididymal weight, n=6-10. M) PCoA, n =3. N) Genus classification horizontal histogram. Summary data are presented as the mean ± SEM. Statistical significance was determined using one-way analysis of variance (ANOVA) followed by Dunnett's multiple comparison test for group comparisons. ns, not significant, * *P* <0.05, ***P* < 0.01, ****P* < 0.001, *****P* <0.0001. ICAM-1, intercelladhesion molecule-1. VCAM-1, vascular cell adhesion molecules-1. ZO-1, zonula occludin 1. AB-PAS, Alcian Blue-Periodic Acid Schiff. IHC, immunohistochemical. HE, haematoxylin and eosin. PCoA, Principal coordinate analysis.


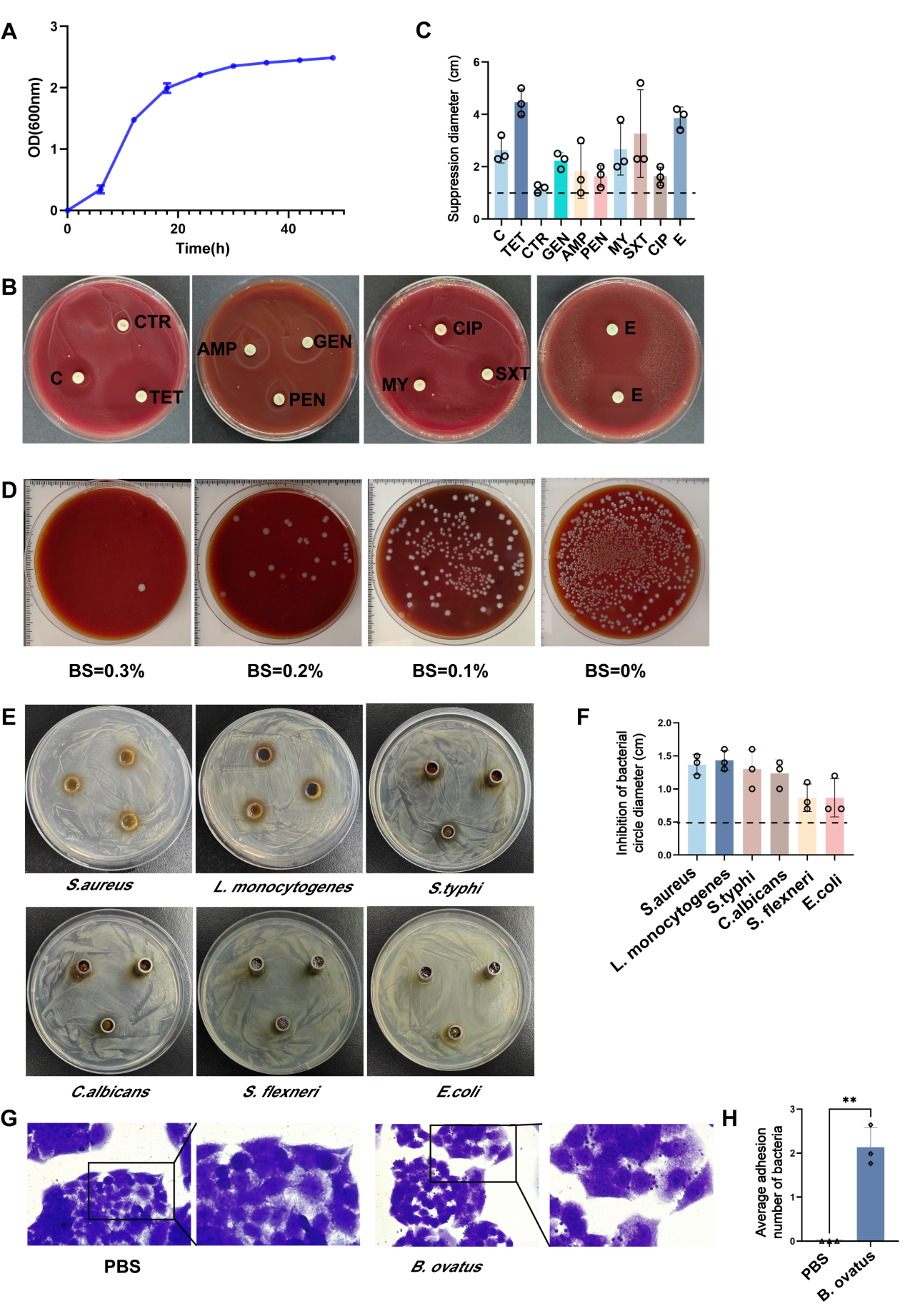


**Figure S3.** A) The survival curve of *Bacteroides ovatus*. B) Drug resistance of *B. ovatus* based on the disk diffusion method. The black dotted line is the diameter of the drug-sensitive paper. C) Suppress diameter quantisation diagram, n = 3 independent experiments. D) Bile salt tolerance test. E) Schematic diagram of the antimicrobial experiment. F) Results of the bacteriostatic experiment. The black dotted line is the Oxford cup diameter, n = 3 independent experiments. G) Adhesion experiments schematic diagram. H) The average number of *B. ovatus* per cell adhesion. Summary data are presented as the mean ± SEM. Statistical significance was determined using Unpaired t-test(H), n = 3 independent experiments. **P* <0.05, ***P* < 0.01. Abbreviations: C, chloramphenicol (30 μg/tablet). CTR, ceftriaxone (30 μg/tablet). TET, tetracycline (30 μg/tablet). GEN, gentamycin (10 μg/tablet). PEN, penicillin (10 μg/tablet). AMP, ampicillin (10 μg/tablet). MY, Lincomycin (2 μg/tablet). CIP, ciprofloxacin (5 μg/tablet). SXT, Compound sulfamethoxazole (25 μg/tablet). E, erythromycin (15 μg/tablet). The tested microbes were: *S. aureus*, *staphylococcus aureus Cowan* 1. *L. monocytogenes*, *listeria monocytogenes* ATCC 19111*S. typh*i, *Salmonella typhi* ATCC 6539. *C.albicans*, *Candid albicans* SC531. *S. flexneri*, *Shigella flexneri* ATCC 12022. *E. coli*, *escherichia coli* O157. BO, *B. ovatus*. BS, bile salt. PBS, Phosphate Buffered Saline.


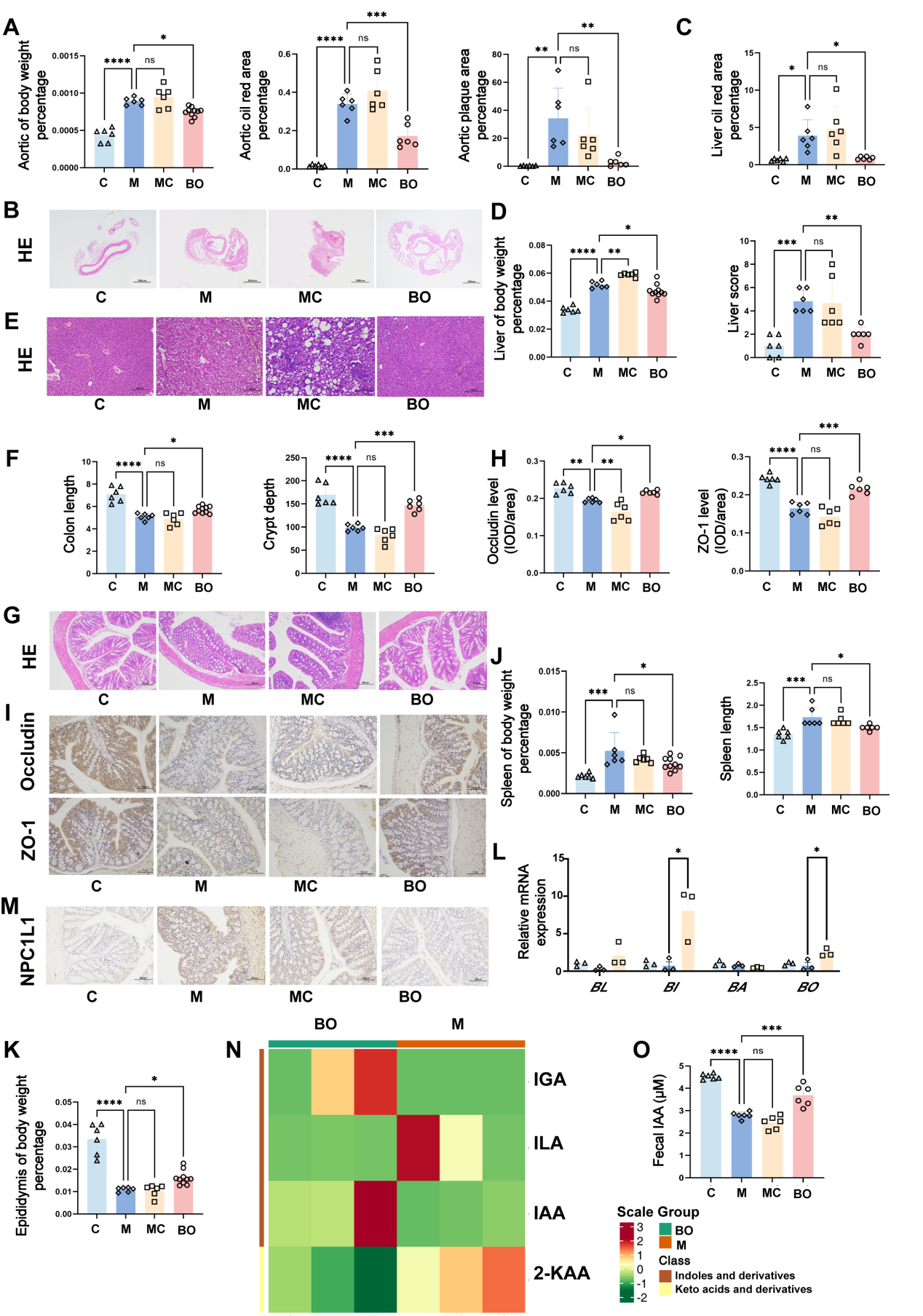


**Figure S4.** A) Aortic gross weight (n=6-10 mice/group), the percentage of the aortic oil red O–stained area (n=6), and the percentage of the aortic HE-stained plaque area (n=6). B) Representative image of HE-stained aorta (Scale bar: 100 μm). C) The percentage of the liver oil red O–stained area, n=6. D) Liver weight (n=6-10), and liver score (n=6-10). E) Representative image of HE-stained liver (Scale bar: 200 μm). F) Quantification of the colon length (n=6-10) and crypt depth(n=6). G) Representative image of HE-stained colon (Scale bar: 200 μm). H) Occludin, and ZO-1 staining in the colon, n=6. I) Representative images of ZO-1 and occludin staining in the colon (Scale bar: 100 μm). J) Spleen weight (n=6-10) and length (n=6). K) Epididymal weight, n=6-10. L) Bacterial abundance in fecal samples based on a quantitative polymerase chain reaction, n = 3. M) Representative images of NPC1L1 staining (Scale bar: 100 μm). N) Differential tryptophan heat map. Red represents a positive correlation, and green represents a negative correlation, n = 3. O) Faecal IAA levels, n=6. Summary data are presented as the mean ± SEM. Statistical significance was determined using one-way analysis of variance (ANOVA) followed by Dunnett's multiple comparison test for group comparisons. n=6-10 mice/group. ns, not significant, * *P* <0.05, ***P* < 0.01, ****P* < 0.001, *****P* <0.0001. Abbreviations: BL, *lactobacillus*, BI, *Bifidobacterium*. BA, *Bacteroides.* BO, *Bacteroides ovatus*. IGA, 3-Indoleglyoxylic acid. IAA, indole-3-acetic acid. ILA, Indole-3-lactic acid. 2-KA, 2-Ketoadipic acid. HE, haematoxylin and eosin. ZO-1, zonula occludin 1. NPC1L1, Niemann–Pick type C1-like 1 protein.


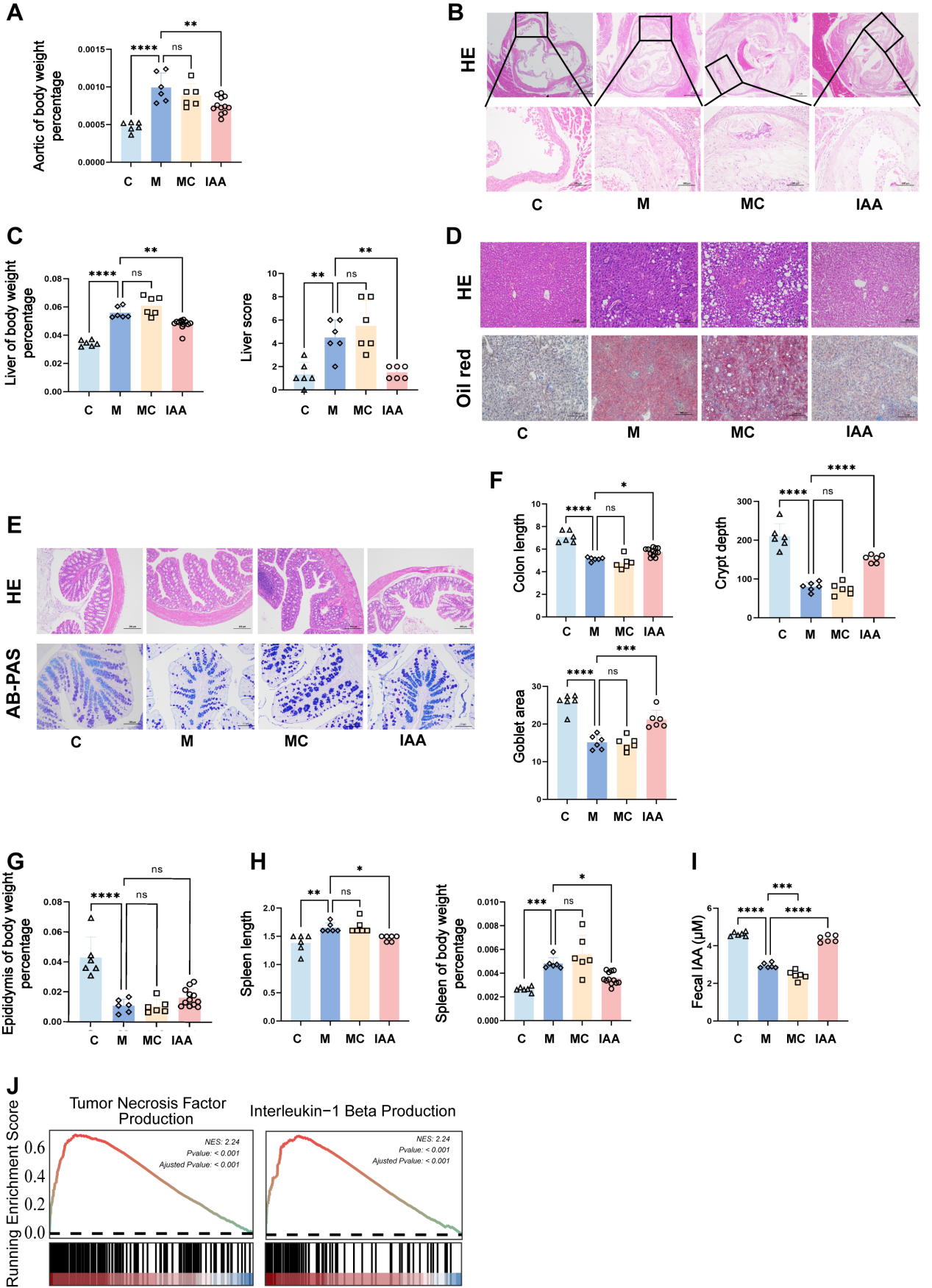


**Figure S5.** A) Gross aortic weight, n=6-12 mice/group. B) Representative image of HE-stained valve (Scale bar: 500 and 100 μm). C) Liver weight (n=6-12), and liver score (n=6). D) Representative image of HE-stained liver (Scale bar: 200 μm). E) Representative image of HE-stained colon (Scale bar: 200 μm) and AB-PAS staining (Scale bar: 100 μm). F) Colon length quantification (n=6-12), and crypt depth (n=6), and the number of goblet cells(n=6). G) Epididymal weight, n=6-12. H) Spleen length (n=6) and weight (n=6-12). I) Faecal IAA content, n=6. J) Control versus model Gene ontology enrichment analysis bubble diagram, showing biological processes (BP), n=3. Summary data are presented as the mean ± SEM. Statistical significance was determined using one-way analysis of variance (ANOVA) followed by Dunnett's multiple comparison test for group comparisons. ns, not significant, * *P* <0.05, ***P* < 0.01, ****P* < 0.001, *****P* <0.0001. Abbreviations: IAA, indole-3-acetic acid. HE, haematoxylin and eosin. AB-PAS, Alcian Blue-Periodic Acid Schiff.


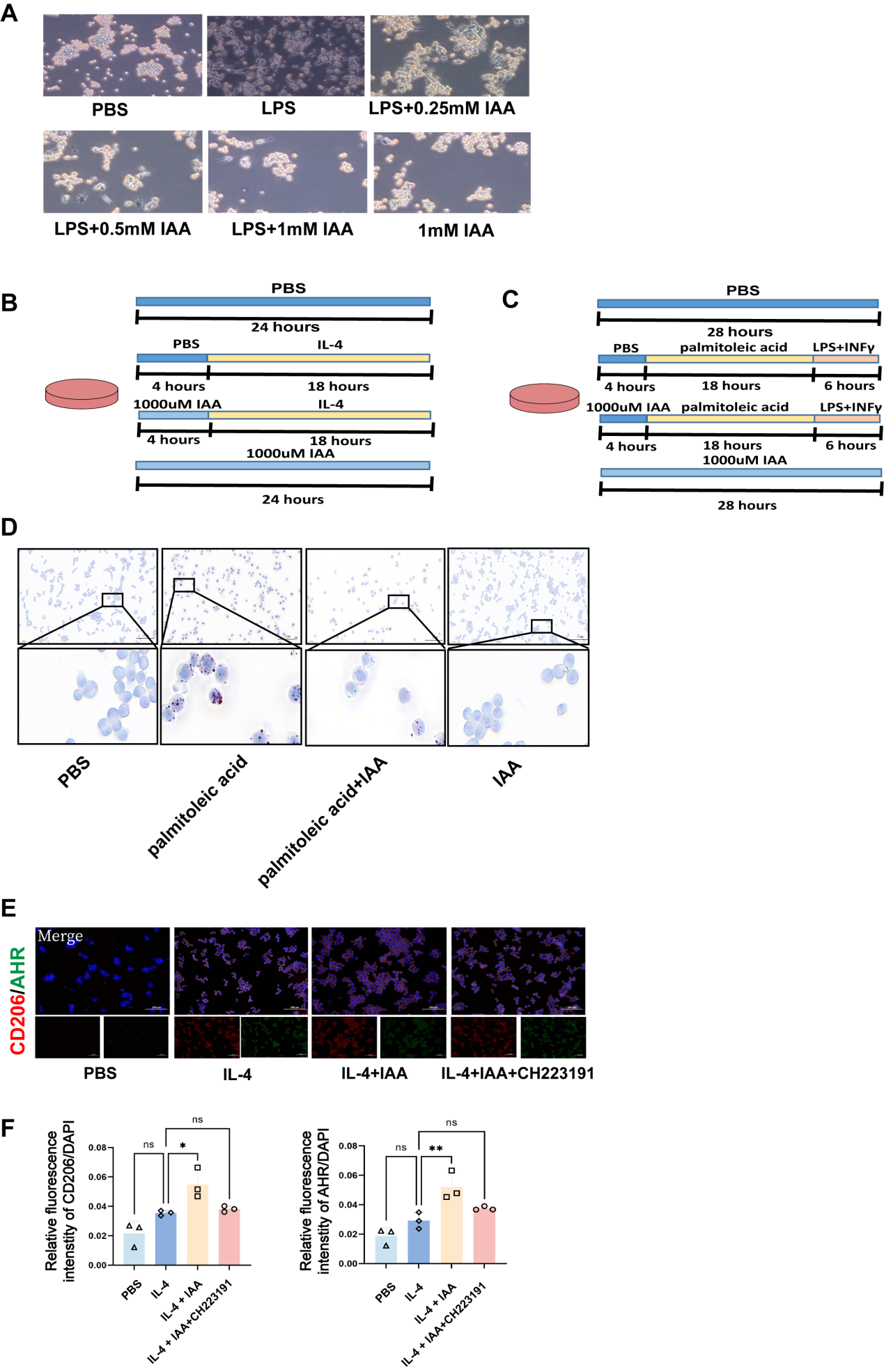


**Figure S6.** A) Representative image of M1 polarisation of RAW264.7 cells after treatment with IAA (Scale bar: 100 μm). B) Experimental design: M2 polarisation was induced with IAA pretreatment for 4 hours, followed by the addition of interleukin 4 (IL-4) for 18 hours. C) Experimental design: a high-fat model was induced with IAA pretreatment for 4 hours, followed by palmitic acid for 18 hours, and finally lipopolysaccharide (LPS) and interferon-gamma (INFγ) for 6 hours. D) Representative image of the oil red O-stained high-fat RAW2264.7 model (Scale bar: 100 μm). E) Representative image of CD206 and aryl hydrocarbon receptor (AHR) staining (Scale bar: 100 μm). F, Relative fluorescence intensity of CD206 and AHR (n = 3 independent experiments). Summary data are presented as the mean ± SEM. Statistical significance was determined using one-way analysis of variance (ANOVA) followed by Dunnett's multiple comparison test for group comparisons. ns, not significant, * *P* <0.05, ***P* < 0.01. Abbreviations: LPS, lipopolysaccharide. IAA, indole-3-acetic acid. IL-4, inteleukin-4.
